# Promoting Positive Youth Development with Adolescent Boys in UK Schools: A Theory-Driven Evaluation of the “Becoming a Man” Programme

**DOI:** 10.1007/s10935-025-00888-1

**Published:** 2025-12-08

**Authors:** Finlay Green, Cristina Preece, Kate Allen, Sean Manzi, Julia Mannes, Lynne Callaghan, Vashti Berry, Ediane Santana de Lima, Amy Woodburn, Tim Hobbs, Julie Harris, Nick Axford

**Affiliations:** 1 Dartington Service Design Lab, Devon, UK; 2 University of Exeter, South Cloisters, St Luke’s Campus, Exeter, EX1 2LU UK; 3 University of Cambridge, Cambridge, UK; 4 University of Plymouth, Plymouth, UK; 5 Cambridgeshire and Peterborough NHS Foundation Trust, Cambridge, UK

**Keywords:** Contribution analysis, Evaluation, Masculinity, Prevention, Violence, Youth

## Abstract

Youth violence, including gender-based violence, is a major public health issue in the UK, yet evidence on effective interventions in the UK remains limited. Becoming a Man (BAM) is a two-year school-based positive youth development programme for adolescent boys, developed in Chicago, US, where two RCTs found reduced violent crime arrests and improved academic engagement. This study evaluated BAM’s potential in the UK using a theory-driven design (no comparison group). 97 boys aged 12–14 years (66% Black/Black British) in three London schools enrolled in BAM. Data sources included: implementation records (recruitment, attendance, adherence, quality, youth socio-demographics); school data (attendance, exclusions, attainment); 36 qualitative interviews (11 scholars, 14 parents, 3 school staff, 3 BAM counsellors (2 interviews each), 1 each from delivery organisation and intervention developer); and a counsellor focus group. Interviews focused on implementation, outcomes and BAM contribution to outcomes. Quantitative data were analysed using descriptive statistics, and qualitative data were analysed using framework analysis. Case studies of 11 scholars explored what worked, for whom, under what circumstances and why. Implementation outcomes were mixed. Attendance targets were met but those for group size, reach and curriculum progression were not. Case study scholars who engaged deeply with BAM’s values demonstrated improved self-control and decision-making, although external influences also affected their development. Concerns about negative labelling were mitigated through balanced recruitment. Limited adverse effects on academic attainment were reported. BAM has potential in the UK but requires further adaptation, implementation support and evaluation (including impact on gender norms).

**Supplementary Information:**

The online version contains supplementary material available at 10.1007/s10935-025-00888-1.

## Introduction

Youth violence is a critical public health issue in the UK, with 24% of 13–17-year-olds reporting recent violence exposure (Youth Endowment Fund, 2023). It is associated with poor mental and physical health (Wright et al., 2017). Key risk factors include poor self-regulation, low self-esteem, social-cognitive deficits, weak parental attachment, school disengagement and disadvantaged neighbourhoods (Lammy, 2017). Protective factors such as educational engagement and cognitive development mitigate risks (Resnick et al., 2004; Ttofi et al., 2016). Youth violence includes gender-based violence (GBV), covering physical, sexual or emotional violence rooted in gender and sexual inequality. This has multiple adverse social and health consequences for victims and perpetrators (Farmer et al., 2023).

While there is considerable evidence on what works to prevent and intervene early to address youth violence (Farrington et al., 2017; Russell, 2021; Kovalenko et al., 2022), interventions proven to be effective in the UK are limited (Youth Endowment Fund, 2020). To address this, the Youth Endowment Fund was established by the UK Government in 2019 to find out what works to prevent youth violence in the UK and build a movement to put this knowledge into practice. One of the interventions it invested in was Becoming a Man (BAM), developed by Youth Guidance (YG) in Chicago. BAM is a school-based positive youth development programme for adolescent boys that fosters social-emotional skills to promote responsible decision-making, including reduced involvement in violence and improved school engagement.^1^ It also challenges boys to reflect critically on their values and actions concerning women. Two randomised controlled trials (RCTs) involving over 4,800 students in Chicago reported significant reductions in violent crime arrests and academic improvements (Heller et al., 2013; 2017), prompting interest in BAM’s applicability to the UK.

While they lack evidence on their effectiveness when implemented in UK contexts, numerous interventions exist for adolescents aimed at preventing violence. These include school-based bullying and dating violence prevention and social-emotional learning programmes, as well as one-on-one youth mentoring (Gaffney et al., 2021; Raposa et al., 2019; Maxwell & Corliss, 2020; Farmer et al., 2023; Chawla et al., 2024). BAM is unusual in adopting a school-based group mentoring approach and targeting boys only. This reflects an appreciation that young males are disproportionately affected by youth violence as both victims and perpetrators (Youth Endowment Fund, 2024). Various factors account for boys and men being disproportionately likely to engage in violence. While higher levels of testosterone in males are associated with aggressive and dominance-seeking behaviour, socialisation and environmental factors are arguably more important. Specifically, traditional social norms associate masculinity with toughness, risk-taking, the suppression of emotions besides anger and aggression, the legitimisation of violence, and dominance over and hostility towards women (Jewkes et al., 2015; Pérez-Martínez et al., 2023). These contrast with the emergence of more positive expressions of masculinity as non-violent and empathetic (Pérez-Martínez et al., 2023). The promotion of positive male social influences and role models is increasingly seen as crucial to efforts to prevent violence among adolescent boys.

Efforts to prevent GBV and other forms of male violence, whether with mixed or male-only populations, are therefore increasingly ‘gender transformative’, meaning that they seek to foster gender-equitable attitudes, behaviours and community structures (Jewkes et al., 2015; Casey et al., 2018; Kato-Wallace et al., 2019). Evidence suggests that such approaches are promising in terms of supporting attitude and behaviour change among men (Casey et al., 2018). While programmes with this emphasis exist in the UK, they are not prevalent and tend not to focus exclusively on boys. Examples include the Mentors in Violence Prevention peer mentoring programme and Equally Safe at School whole school approach. There is therefore a need for evaluations in the UK of programmes for boys with a gender-transformative emphasis, such as BAM.

Building on a successful feasibility phase in the period 2020–2022 (Green et al., 2023a), this pilot study evaluated BAM’s implementation in three schools in London, UK. Its aims were to: (i) assess implementation feasibility, (ii) establish evidence of promise and (iii) inform a next-stage evaluation design. We examined BAM’s programme theory to establish evidence of promise, including potential unintended consequences.

The theory of change (ToC) was developed with Youth Guidance (YG) and the Mental Health Foundation (MHF) in 2020 (Supplementary File 1). The ToC comprises four sub-theories: implementation, intermediate outcomes, ultimate outcomes and unintended consequences. BAM aims to promote responsible decision-making, including improved educational attainment and reduced involvement in violence (the ultimate outcomes). This is achieved by helping participants – known as ‘scholars’ – internalise six core values (the intermediate outcomes – integrity, self-determination, positive anger expression, accountability, respect for womanhood, visionary goal-setting), which serve as protective assets. Scholars’ ability to apply these values depends on wider influences in their lives that may reinforce or limit their efforts. Internalisation occurs through high-quality delivery of BAM with fidelity to the intended target population, who attend regularly. This helps scholars actively experience and reflect on the core values. Their engagement depends on their readiness to form healthy group dynamics marked by fun, safety, openness and mutual challenge. However, implementation may also produce unintended consequences, such as negative group labelling or fractious group dynamics, which risk motivating anti-social behaviour.

The ToC and its four sub-theories guided the pilot evaluation’s research questions (RQ):

1. To what extent is BAM being successfully implemented, with whom, under what circumstances and why?
2. To what extent is implementation contributing to improvements in young people’s social-emotional development, for whom, under what circumstances and why?
3. To what extent are improvements in social-emotional development contributing to improved academic engagement and reduced involvement in crime/anti-social behaviour, for whom, under what circumstances and why?
4. To what extent has implementation contributed to unintended consequences, for whom, under what circumstances and why?

While quantitative outcome data collection and analysis were integral to the pilot (see Methods, also Green et al., 2024), the absence of a comparison group limits their significance. Therefore, this article focuses on BAM’s potential contribution to observed outcomes ascertained primarily through qualitative methods.

## Methods

### Overview

We used a non-randomised, theory-driven design (no comparison group). The study took place in three schools in London (2021–2023). 97 boys aged 12–14 years (66% Black/Black British) enrolled in BAM. Data sources included: implementation records (recruitment, attendance, adherence, quality, youth socio-demographics); school data (attendance, exclusions, attainment); 36 qualitative interviews (11 scholars, 14 parents, 3 school staff, 3 BAM counsellors (2 interviews each), 1 each from delivery organisation and intervention developer); and a focus group with counsellors. Interviews focused on implementation, outcomes and BAM contribution to outcomes. Quantitative data were analysed using descriptive statistics, and qualitative data were analysed using framework analysis. Case studies of 11 scholars combined realist evaluation and contribution analysis to explore what worked, for whom, under what circumstances and why. Methods and results are summarised here using the CONSORT extension for pilot and feasibility studies (Eldridge et al., 2016), excluding non-applicable items (Lancaster & Thabane, 2019) (Supplementary File 2).

### Intervention

BAM is delivered in the UK by the Mental Health Foundation (MHF) alongside two London-based community organisations: Black Thrive and Colourful Minds. BAM promotes social-emotional learning to enhance school engagement and reduce involvement in the criminal justice system. The intervention is outlined in full in the TIDieR checklist (Hoffmann et al., 2014) (Supplementary File 3).

BAM targets adolescent boys who face challenges with social-emotional development. Scholars are initially identified by school staff and BAM counsellors (the intervention’s practitioners) based on factors such as risk of exclusion, poor academic performance or mental health concerns. A two-to-three-month orientation period allows counsellors to invite scholars to participate, after which scholars decide whether to continue. At three to five months, scholars complete the Holistic Student Assessment (HSA; Allen et al., 2017), not to determine continued participation but to assess recruitment effectiveness. The HSA is a 61-item self-report tool that provides a portrait of a young person’s long-term social-emotional development, grouping scholars into three tiers: primarily strengths and few challenges (Tier 1); mixed strengths and challenges (Tier 2); or primarily challenges (Tier 3). BAM seeks a balanced representation across all three tiers, with the majority of scholars from Tier 2. This structure helps prevent BAM from being perceived solely as a programme for “bad students”.

The programme comprises four activities, all delivered by a BAM counsellor:

1. BAM Circles: Weekly one-hour in-school group sessions for 8–12 scholars, guided by a 30-lesson curriculum (each lesson taking 1–3 sessions), with activities such as role plays, group missions, check-ins and homework.
2. Brief Encounters: Informal, brief check-ins with scholars to maintain engagement and support development.
3. Special Activities: Out-of-school group events for scholars to reinforce learning through team tasks and social interaction, including go-karting and theme park trips.
4. One-to-One Support: Individualised sessions for higher-need scholars, providing emotional support and reinforcing BAM Circles content.

BAM counsellors are recruited from scholars’ local communities, ensuring relatability and shared experiences. They must have experience of working with youth in therapeutic or mentoring roles. They receive 300 h of training and ongoing coaching from MHF and YG to ensure delivery quality and fidelity to the programme model, through adherence to the programme manual – the “BAMual” – that contains the curriculum. Three BAM counsellors were recruited and trained during the feasibility and pilot phases.

### Overarching Approach

Evaluations using ToCs are often criticised for not testing the causal logic of interventions fully (Breuer et al., 2016). Realist evaluation addresses this by generating context–mechanism–outcome (CMO) hypotheses to explore “what works, for whom, under what circumstances, and why” (Pawson & Tilley, 1997). ToCs complement this by providing a holistic, visual representation of the intervention’s logic, which engages stakeholders and helps prioritise key elements for testing (Blamey & Mackenzie, 2007; Rolfe, 2019). This study combined both approaches to articulate the mechanisms and contextual factors underpinning BAM’s programme theory.

The ToC (Supplementary File 1) drew on previous iterations developed by YG, academic literature, stakeholder expertise, intervention documents and observations of BAM delivery in Chicago. Sources included BAM research (Lansing & Rapoport, 2016; Heller et al., 2013, 2017), frameworks for group therapy (Yalom & Leszcz, 2005), psychotherapy (Jung, 1969), youth development research (Nagaoka et al., 2015; Bonell et al., 2016) and implementation science models (Moore et al., 2015; Proctor et al., 2011).

Contribution analysis was applied to assess BAM’s contribution toward improved outcomes. This was chosen for its ability to: (i) systematically assess the contribution of interventions to results (Lemire et al., 2012), addressing challenges seen in realist evaluations (Marchal et al., 2018) and ToCs (Breuer et al., 2016); and (ii) consider rival influences, often overlooked in theory-based evaluations (Breuer et al., 2016).

Contribution analysis triangulates data from multiple sources to assess how an intervention contributes to specific outcomes (Mayne, 2001). Stakeholders iteratively review the evidence to build a “plausible contribution story” (Mayne, 2012). Building on Mayne (2012), the pilot study’s contribution story would be deemed plausible if it met three criteria. The first, implementation, refers to whether BAM’s activities aligned with the ToC. Targets were set for recruitment, attendance and adherence, guided by YG’s benchmarks and published research on BAM (Heller et al., 2013; 2017). They were considered alongside accompanying evidence regarding with whom BAM was successfully implemented, under what circumstances and why. The second criterion, outcomes, was adapted to reflect the pilot nature of the study. Rather than assessing whether the ToC was supported across the full cohort, we examined whether and how the expected results chain played out across a series of embedded case studies, further detail on which is provided below. This involved assessing the extent to which improvements in social-emotional development and responsible decision-making were observed in individual cases. We considered whether the assumptions underlying each link in the ToC were met, and whether these links held under different contextual conditions, while also accounting for alternative explanations. The third criterion considered unintended consequences, specifically examining evidence that either (i) no adverse effects materialised or (ii) any that did were recognised and their impact was deemed manageable.

### Participant Selection

The evaluation included scholars, their parents, BAM counsellors and staff from participating organisations (MHF, YG, schools). At the start of the programme (October 2021), MHF gained informed consent from all parents and scholars to participate in service evaluation activities (ongoing, routine monitoring activity concerned with current service activity) (Twycross & Shorten, 2014).

At the start of BAM, 71 parents consented to MHF sharing their contact details with the evaluation team. Researchers contacted each parent via text and email, arranging a suitable time to discuss participation and schedule an interview. Parents who agreed to be interviewed also provided consent for their child to participate in a separate interview. Informed consent was then obtained directly from the scholar. Parent interviews were conducted by phone, while scholar interviews took place in person at their schools.

### Data Collection

The ToC was divided into specific elements and organised in a data collection table, based on Funnell and Rogers’ (2011) approach. This table outlined methods, data sources, targets and participants, enabling thorough data collection. A sub-section is provided in Supplementary File 4. Both quantitative and qualitative data were collected.

Starting with quantitative data, all implementation data were gathered through MHF’s routine monitoring, covering the following domains: (i) Recruitment – retention and the percentage of scholars meeting eligibility criteria based on the HSA; (ii) Attendance – counsellor-completed records of the number of BAM Circles delivered and the length of sessions; (iii) Adherence – counsellor-completed checklists following group sessions and assessments of counsellors’ key competencies by the Replication Specialist^2^; and (iv) Quality – appraisals of counsellors’ performance by the Replication Specialist against eight key competencies through observation and insights from training and coaching (Supplementary File 5). These implementation outcomes were informed by frameworks from Carroll et al. (2007) and Proctor et al. (2011). Cost and sustainability were beyond this study’s scope. Acceptability and participant responsiveness were evaluated based on participants’ interaction with key programme mechanisms, given their conceptual overlap. MHF also collected socio-demographic information on scholars and counsellors.

Qualitative data collection comprised 36 individual interviews (11 scholars, 14 parents, two interviews with each of the three counsellors, three school staff, one YG Replication Specialist, one MHF Project Manager) and one focus group with counsellors. All sessions were audio-recorded and transcribed verbatim. We used purposive sampling (Patton, 2015) guided by the ToC. We applied realist interview principles by grounding each topic guide in the relevant elements of the ToC (Manzano, 2016) (see Supplementary File 6 for an example). We remained open to further unintended consequences throughout.

Each of the 11 scholars we interviewed was treated as an embedded case study (Yin, 2018), meaning they were considered sub-cases within the larger case under investigation (the BAM programme). A common challenge in theory-driven evaluations is that individual elements of the programme theory can become disconnected, with more emphasis placed on the elements themselves rather than on the relationships between them (Pawson & Manzano-Santaella, 2012). By protecting the integrity of sub-cases, embedded case studies enabled us to retain a focus on these connections in our programme theory, centring the journey of each scholar through the programme. Rigour within embedded case studies is understood through the degree to which triangulated sources converge or diverge. To ensure comprehensive data on each scholar’s journey, we also asked counsellors, school staff and parents about the young person’s experience.

### Methods Learning from the Feasibility Phase

Iterating data collection methods is crucial for enhancing feasibility and pilot study quality (O’Cathain et al., 2015). During the feasibility phase, feedback from MHF, YG and scholars suggested using interviewers with shared lived experiences (the first round was conducted by White female researchers) and returning to in-person interviews with scholars (after remote interviews due to the pandemic), making them more creative. In response, nine of the 11 scholar interviews in round two were conducted by two female researchers from racially minoritised backgrounds. Additionally, and based on Macedo (2022), second-round interviews used a hot air balloon activity to reflect on scholars’ BAM journeys (Supplementary File 6). Scholars were invited to describe how they felt at the start and end of BAM, what propelled them forward (wind), who supported them (basket) and obstacles or supports encountered (stormy/sunny weather). Emoji cards and prompts encouraged reflection, fostering an interactive and less formal setting. This offered several benefits: (i) engaging scholars in a fun, reflective activity; (ii) creating a relaxed environment, reducing the pressure of direct questioning; (iii) enabling consideration of broader influences on scholars’ experiences, supporting contribution analysis; and (iv) simplifying discussions on mechanisms (wind) and context (basket, weather).

### Analysis

**Fig. 1:**
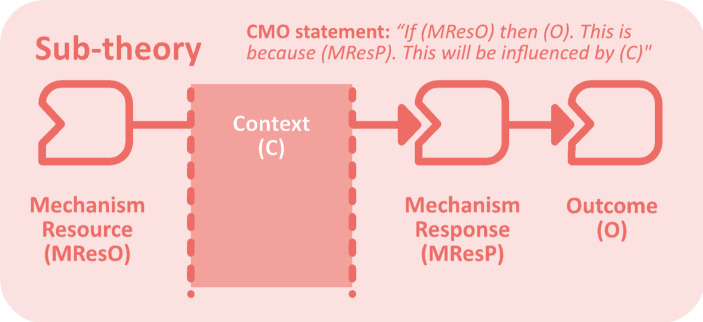
Realist matrix structure of each sub-theory, using the amended CMO framework proposed by Dalkin et al. (2015)

In evaluations grounded in contribution analysis, the contribution story that summarises the findings comprises a series of contribution claims, each grounded in evidence. We constructed contribution claims for our four sub-theories – implementation, intermediate outcomes, ultimate outcomes, unintended consequences – each addressing a specific research question, each summarising how much of the ToC was verified, considering other influencing factors (Mayne, 2012). The contribution claims for intermediate and ultimate outcomes focused on the validation of the ToC among the 11 embedded case studies, rather than the cohort as a whole. The sub-theories were structured as realist matrices (Ebenso et al., 2019) in which different combinations of contextual factors, mechanisms and outcomes are grouped under a higher-order CMO statement. We adapted Dalkin et al.’s (2015) framework to distinguish between mechanism resources and responses, clarifying how programme strategies lead to outcomes (Fig. 1). We adapted Delahais and Toulemonde’s (2012) approach to contribution claims to account for the four components within each sub-theory: mechanism resource, mechanism response, context, and outcome. Each claim assessed whether: (i) the outcome (O) occurred… (ii) due to the mechanism response (MResP)… (iii) which was driven by the mechanism resource (MResO)… (iv) mediated by context (C)… (v) while accounting for rival influences (R).

Both quantitative and qualitative data were analysed within this overarching approach. Quantitative data were analysed using descriptive statistics, covering recruitment, attendance, adherence, quality and scholars’ socio-demographic characteristics. Qualitative data were analysed using framework analysis (Ritchie & Spencer, 1994; Brand et al., 2019; Gale et al., 2013), a deductive method suitable for researchers with predefined ideas of how to interpret the data (guided here by the ToC). A ‘framework matrix’ was developed in Microsoft Excel, with columns representing elements of the ToC and rows representing individual participant data (e.g., a parent interview). Each transcript was coded into this matrix, with relevant evidence placed in each cell (Brand et al., 2019). Certainty was assessed by the degree of alignment between evidence and programme theory (Lemire et al., 2012). Insights from quantitative data were added to the framework matrix to apply an integrated approach during synthesis.

Following Delahais and Toulemonde’s (2012) guidance, we conducted a triangulation assessment to inform the contribution claims. Using Campbell et al. (2020) and Farmer et al. (2006), convergence was assessed at three levels: among individuals, across stakeholders and across methods. In Results, ‘strong’ evidence is defined as full or fundamental convergence at two or more levels of triangulation, such as between individuals (e.g., parents), across stakeholders (e.g., parents, scholars), or across methods (e.g., interviews, programme data).

## Results

**Fig. 2:**
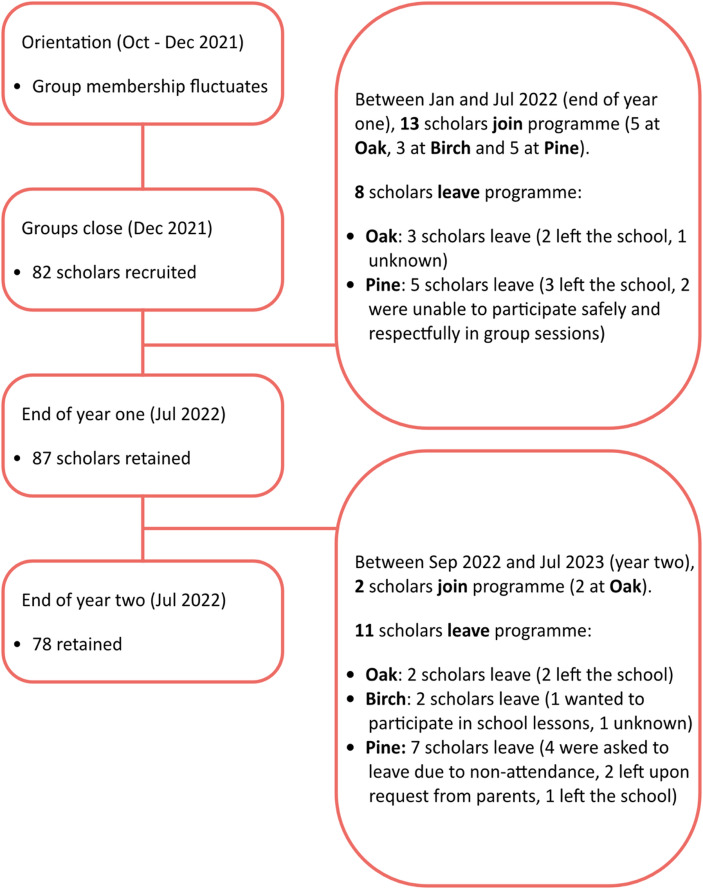
Number of scholars recruited, lost through attrition and retained

Of 97 scholars enrolled in BAM in October 2021, 78 (80%) remained by July 2023 (Fig. 2). Fifty-five of the 97 scholars (57%) attended Pine^3^, which was hosting its first BAM cohort, allowing the counsellor to form five groups at the start of the pilot phase. Oak and Birch, having participated in the feasibility phase, each added two new groups alongside the four established in the previous phase.

All 97 intervention participants were part of the service evaluation activities undertaken by MHF. These activities generated the evidence reported below concerning implementation data on scholar recruitment, attendance and adherence, scholars’ background characteristics and baseline data from the HSA on the level and spread of need in the pilot phase cohort. The demographic characteristics of intervention participants (all boys) are shown in Table 1. The vast majority (89%) were in Years 8 and 9 (12–14 years) at entry and two-thirds (66%) were Black/Black British. Rates of free school meal eligibility and English as an Additional Language were higher than the national average in all three schools, while rates of students needing support with special educational needs or disabilities (SEND) were higher than the national average in two schools (Table 2).

**Table 1:** Participant characteristics

School year	% (N = 97)
8	37
9	52
10	10
11	1

Ethnicity	% (N = 97)
Black/Black British	66
Mixed ethnicity	19
White/White British	10
Other	3
Missing	2

**Table 2:** Rates of free school meal eligibility, special educational needs and English as an additional Language in participating schools*

Additional	Oak (%)	Birch (%)	Pine (%)	National average (%)
Eligible for FSM	43.6	38.1	52.8	23.8
With EHC Plan	4.0	7.8	3.6	4.3
Requiring SEND support	20.0	17.8	13.0	13.0
With EAL	51.2	60.4	51.5	20.2

*FSM Free school meals,* EHC* Education, health and care,* SEND* Special educational needs or disabilities,* EAL* English as an additional language

Results for each research question are reported below (summarised in Box 1). In reporting the results from qualitative data we use verbal markers to indicate the number of scholars and parents sharing particular views, following common research practices (Maxwell, 2010; Sandelowski, 2001); ‘most’ refers to the majority, while ‘some’ refers to less than half.

### RQ1: The Extent to Which BAM was Implemented Successfully

This section presents findings on implementation support for counsellors and key implementation outcomes. We draw on the triangulation assessment to examine how a positive learning environment – facilitated by implementation support – contributed to these outcomes. This assessment, a core element of our approach to contribution analysis, integrated evidence across individuals, stakeholder groups and data sources to assess the validity of the ToC components and causal links relevant to RQ1.

**Table:** 

**Box 1: Summary of Contribution Claims**
*RQ1: To what extent is BAM being successfully implemented, with whom, under what circumstances, and why?* There is strong evidence that the implementation of BAM varied by implementation domain (O). Adaptation and delivery quality were generally successful, but recruitment and attendance faced challenges. While counsellors adhered well to the manual, curriculum progression was limited. Counsellors’ prior skills supported delivery (R), but implementation support from MHF and YG (MResO) played a crucial role in creating a positive learning environment for counsellors (MResP). This environment significantly contributed to implementation, although the contributions of peer supervision, project management support and community partners were limited due to capacity and resource limitations. Incompatible school contexts and counsellor absences also negatively impacted implementation (C).
*RQ2: How has implementation contributed to any reported changes in social-emotional and identity development, for whom, under what circumstances, and why?* There is strong evidence from qualitative data that BAM made an important contribution to case study scholars’ development. While BAM was not seen as the primary contributor, ranking second or third behind family, friends, faith or football (R), the counsellors and curriculum (MResO) played a significant role. By fostering action and reflection, they helped scholars improve emotional control and develop a stronger sense of self (MResP). Case study scholars’ readiness and willingness to follow group conditions – having fun, being safe and challenging themselves – along with wider life influences, shaped their engagement (C).
*RQ3: How has social-emotional development contributed to any reported changes in academic performance and behaviour, and the avoidance of, or reduced involvement in crime/anti-social behaviour, for whom, under what circumstances, and why?* There is strong evidence that most case study scholars were applying themselves more at school and making responsible decisions in risky situations (O). While other sources of support or motivation beyond BAM contributed to responsible decision-making (R), the core values made a significant impact (MResO), empowering scholars to seize opportunities and protecting them from risks (MResP). In some instances, wider influences in scholars’ lives reinforced their commitment to positive choices, while in others these influences added pressure and challenges (C).
*RQ4: How has implementation contributed to any reported unintended consequences, for whom, under what circumstances, and why?* There is mixed evidence on whether BAM inadvertently encouraged irresponsible decision-making. Strong evidence indicates that negative labels did not develop within BAM groups. Although factions were present, they did not consist of students widely regarded as disruptive within the broader school community, reducing the risk of negative labelling. The impact of missing lessons on academic attainment yielded mixed evidence. None of the counsellors, scholars or parents interviewed reported negative effects but some school staff expressed concerns, particularly for scholars receiving multiple school-based interventions.
With EAL

*FSM Free school meals,* EHC* Education, health and care,* SEND* Special educational needs or disabilities,* EAL* English as an additional language

### Implementation Support

The evidence on the effectiveness of counsellors’ implementation support, referred to as ‘backbone support’ by the counsellors, was mixed. Training and coaching emerged as key contributors to fostering a positive learning environment, the main driver of implementation outcomes hypothesised in our ToC. However, the roles of peer supervision, project management support and community partners were less significant.

Due to the COVID-19 pandemic and the Replication Specialist being based in Chicago, curriculum training was delivered online in shorter, more frequent sessions than usual, with video run-throughs and peer feedback. The full coaching model, including goal setting, observation and feedback, was implemented as planned. By the end of the pilot phase, two of three counsellors ranked training and coaching as their most significant support and the biggest contributor to a positive learning environment, while the third ranked it second. Counsellors valued the flexible, personalised approach, with appreciation increasing in year two due to three factors. First, MHF expanded BAM to three additional schools in London in Autumn 2022, resulting in fewer group training sessions and more individualised coaching for counsellors, as the two teams (original and new) progressed through the curriculum at different rates. Second, counsellors received USB sticks with online training materials, including video walkthroughs developed by YG during the pandemic. Third, with COVID-19 restrictions lifted, the Replication Specialist conducted more in-person observations during five visits in year two, addressing concerns about limited in-person oversight in year one.

Two counsellors ranked peer supervision as the second most significant contributor to their development during year two, while one ranked it first. However, all three agreed its quality had declined compared to year one. Scheduling issues, partly due to the MHF BAM Programme Manager’s time constraints, reduced the frequency of peer supervision in year two, negatively impacting counsellors: *“With all these different demands … I don’t feel like it’s institutionalised like it used to be … I do feel affected emotionally.”* (BAM Counsellor).

Counsellors valued project management support, including emotional and practical assistance with recruitment and school-related challenges. However, this support declined in year two due to increased demands. The Programme Manager, tasked with managing counsellors and organising training, faced challenges as the number of counsellors and schools doubled, recruitment issues arose in new London sites, and the MHF team’s capacity shrank. While all counsellors appreciated the trust placed in them, they, the Replication Specialist and the Programme Manager agreed that more proactive and timely school support was needed.

Regarding community partner support, Black Thrive and Colourful Minds contributed to adapting the BAMual and providing training on anti-racism. However, resource constraints limited their overall involvement according to counsellors and school staff.

#### Implementation Outcomes

The evidence on BAM’s feasibility in its new context is mixed. Starting with *adaptation*, the positive learning environment in which the counsellors operated played a key role in adapting BAM to the UK. This collaborative process facilitated changes to both the curriculum and implementation support (Green et al., 2023b).

Moving onto *recruitment*, Table 3 summarises the extent to which BAM met its targets for recruitment, attendance and adherence. While some targets were met or exceeded, others were not. 55 of 61 scholars who completed the HSA presented with at least one emotional well-being challenge. While Birch and Pine demonstrated a spread of need across all three HSA tiers, Tier 3 scholars were overrepresented and Tiers 1 and 2 were underrepresented compared to the indicative target 15/70/15 split. At Oak, the cohort was skewed towards higher-need scholars, with no Tier 1 scholars and 56% meeting Tier 3 thresholds. Only half the groups included representation from all tiers (Table 4).

**Table 3:** Summary of findings regarding implementation outcomes

Implementation area	Target	Target met?
1. Recruitment	Each counsellor has groups of eight-to-12 scholars by January 2022.	**Partially met**. Six out of nine groups reached the target size.
	All scholars are experiencing challenges in at least one area of their social-emotional development, according to the HSA	**Partially met**. 55 of 61 scholars who completed the HSA had at least one challenge related to emotional well-being.
	All three tiers of need are represented in each group and school according to the HSA, with an indicative target split of ~ 15%/70%/15% for Tiers 1, 2 and 3, respectively*.	**No**. All three tiers represented in Birch and Pine but not Oak, which only had students from tiers 2 and 3. Four out of eight groups only had students from tiers 2 and 3.
2. Attendance	Scholars attend a minimum of 13 out of an intended 25 BAM circle sessions on average every school year.	**Yes**. Scholars attended an average of 17 and 13 sessions in years one and two.
	Every scholar receives at least one brief encounter a month during term time.	**No**. Target met in one month only at Oak and not at all at Birch or Pine.
3. Adherence	Counsellors deliver all lessons from the 30-lesson BAM Manual (roughly two sessions per lesson) with each group.	**No.** No group progressed past lesson 23.
	Counsellors deliver 25 BAM Circles per year with each group.	**No.** Five out of eight groups met the target during year one and four in year two, all at Pine.

*YG has since revised the indicative target to 25/50/25, as the original 15/70/15 split was found to be impractical in most schools, where Tier 1 scholars typically outnumber those in Tiers 2 and 3. The revised distribution is considered sufficient to support and sustain the intended group conditions

**Table 4:** Percentage of scholars in each tier of the Holistic Student Assessment (HSA) within each group, and group sizes by December 2021*

School/group	Tier 1 (%)	Tier 2 (%)	Tier 3 (%)	Group size (*N*) by December 2021
Oak (N = 19)				
A	0	44	56	7
B				5
Birch (N = 23)				
C	0	82	18	12
D	14	43	43	8
Pine (N = 55)				
E	13	50	18	9
F	11	33	56	11
G	25	58	17	12
H	0	60	40	12
I	-	-	-	6

*Groups A and B were merged prior to administration of the HSA; the remaining group at Pine (Group I) was formed after the HSA was administered

Despite these challenges, counsellors reported that the recruitment process improved during the pilot phase because of lessons learned in the feasibility phase. At Oak and Pine, counsellors collaborated closely with senior school leadership, heads of year and pastoral staff to identify scholars. However, high overall levels of need at Oak, exacerbated by COVID-19, resulted in a cohort with significant challenges. At Birch, a misunderstanding of BAM’s purpose contributed to an overrepresentation of higher-need scholars. The counsellor felt that school staff viewed BAM as a behaviour management intervention rather than a positive youth development programme, skewing recruitment.

The overrepresentation of higher-needs scholars in the cohort affected group sizes. At Oak, MHF initially planned for one group but split it into two (Groups A and B) in November 2021 because of concerns about group dynamics. The counsellor noted high needs and challenging behaviour, which hindered programme delivery and curriculum progression. At Pine, Group I was formed later at the school’s request, limiting its size. As a result, only six of the nine groups reached the target size of 8–12 scholars by the end of December 2021 (Table 4).

Turning next to *quality of delivery*, counsellors felt their prior skills in listening, assessing and engaging scholars were important. However, as outlined above, convergent evidence from the triangulation assessment – particularly the counsellors’ focus group – indicates that the positive learning environment created by MHF and YG, especially through training and coaching, was crucial to their development.

The Replication Specialist assessed each counsellor against eight key competencies through observation and supervision at the start and end of year two (Supplementary File 6). Ratings of ‘Intermediate’ or ‘Advanced’ indicated that expectations had been met or exceeded, respectively. These expectations were met across all competencies except ‘Challenging and Confronting’, where two counsellors were rated ‘Novice’, and ‘Systemic Leadership’, where one counsellor received the same rating.

At both timepoints, all counsellors were rated either ‘Intermediate’ or ‘Advanced’ in *Clinical Listening and Assessing*, *Youth Engagement* and *Modelling*. These ratings aligned with feedback from scholars and parents, who described the counsellors as empathetic and relatable, with shared experiences fostering trust and engagement. Progress in *Curriculum Fidelity* and *Group Work* was slower: all counsellors started year two at ‘Novice’ and reached ‘Intermediate’ by year’s end. Early challenges in session planning affected *Curriculum Fidelity*, while difficulties in managing group dynamics limited progress in *Group Work*. *Challenging and Confronting* showed the least improvement. Two counsellors remained at ‘Novice’ throughout, and the third made only limited progress. *Men’s Work* also developed slowly: two reached ‘Intermediate’ by year’s end and one remained ‘Novice’. Limited in-person coaching and delayed participation in the Mankind Project Weekend Training Adventure, which supports vulnerability and self-reflection, were noted as barriers to development in both areas. In *Systemic Leadership*, two counsellors achieved an ‘Advanced’ rating due to strong engagement with school staff, while the third remained at ‘Novice’.

Regarding attendance, all scholars attended at least one group session, with average attendance meeting the target of 13 sessions per year (17 in year one, 13 in year two). Counsellors attributed this to implementation support and the engaging and varied sessions BAM offered, which, scholars agreed, offered a positive contrast to the school environment where scholars often felt overlooked. Nonetheless, YG and MHF held ambitions for higher numbers of sessions attended – closer to the 21 sessions reached in the second year of the second Chicago RCT.

Challenges with attendance at group sessions were shaped by scholars’ start dates, retention and group scheduling. Regarding start dates and retention, 15 of the 97 scholars joined late and 19 left early due to reasons such as leaving the school (*n* = 8), parental withdrawal for academic focus (*n* = 3), disruptive behaviour (*n* = 2) or poor attendance (*n* = 4). Consequently, 35% did not complete the programme, and late starters were recruited to fill these gaps. As for group scheduling, five groups met the target of 25 sessions during year one and four in year two (Table 5). All were at Pine. Scheduling required school support, including timetabling, informing teachers and securing space. At Birch, convergent evidence from school staff, the counsellor and the Replication Specialist indicated that limited support, misconceptions about BAM’s perceived permissive approach to behaviour (versus the school’s authoritative stance), staff turnover and reduced Programme Manager capacity disrupted implementation. At Oak, scheduling was disrupted by staff sickness, transport strikes and school closures, while at Pine GCSE^4^ commitments adversely affected Group I’s attendance in year two.

**Table 5:** Number of BAM circles held in each group and year, and number of lessons delivered per year

School/group	BAM circles in Year 1	BAM circles in Year 2	Total BAM circles	Lessons in Year 1 (target 15)	Lessons in Year 2 (target 30)
Oak					
A	22	14	36	8	16
B	20	20	40	9	14
Birch					
C	15	4	19	6	12
D	19	9	28	5	13
Pine					
E	33	25	58	15	21
F	29	25	54	15	23
G	32	26	58	15	23
H	27	26	53	13	19
I	29	11	41	15	22

We also measured attendance in relation to brief encounters and one-to-one support. The target of one brief encounter per scholar per month was not met at Birch or Pine and only once at Oak. Around 10% of scholars were expected to receive one-to-one support, which happened at Pine but not at Oak or Birch. Data on brief encounters was incomplete due to their frequency and short duration, leading to likely under-reporting. At Birch, the counsellor reported that one-to-one delivery was hindered by difficulties withdrawing scholars from lessons, while counsellor absences negatively impacted delivery at Oak. At Pine, regular playground duty by the BAM counsellor provided opportunities for brief encounters and one-to-ones, supplemented by scholars voluntarily visiting the BAM room.

The final implementation outcome was adherence. Counsellors struggled to complete all lessons (Table 5). As a result, the final two core values – Respect for Womanhood and Visionary Goal Setting – were not delivered. While progression through lessons was limited by the difficulties counsellors faced scheduling sessions, smaller groups also posed challenges by rendering some lessons infeasible. The Replication Specialist also felt that the difficulties counsellors faced with the group work competency adversely impacted curriculum progression. Counsellors felt that some of the challenges they faced with group dynamics stemmed from issues with recruitment. All counsellors felt that they faced issues with pre-existing factions in groups, which undermined group safety and limited curriculum progression. These implementation challenges are common in BAM: counsellors in Chicago often struggle to deliver the final two values.

### RQ2: The Extent to Which Implementation Contributed to Improvements in Young People’s Social-Emotional Development

Most of the evidence generated for RQ2 was drawn from the embedded case studies, which captured the perspectives of school staff, counsellors, parents and scholars on the journeys of 11 individual scholars. Triangulated data from these cases indicated that case study scholars who experienced social-emotional and identity development through BAM engaged in its action-reflection cycle: experiencing alignment with core values through activities, then reflecting on these experiences with their counsellors and peers. Due to implementation challenges, only the first four core values – integrity, self-determination, positive anger expression, accountability – were fully delivered, fostering scholars’ growth in these areas.

Counsellors, parents and most case study scholars highlighted that physical activities and freedom to express themselves encouraged engagement. Activities such as boxing and group missions balanced fun with challenge, motivating competitive scholars through goal-setting and rewards. These active learning processes helped scholars to practise the resilience needed to remain aligned with BAM’s first core value, integrity (acting in line with one’s desired self).

Positive anger expression emerged as a transformative area for many case study scholars, particularly those navigating low self-worth or secondary school transitions: by learning to express vulnerabilities constructively, they experienced catharsis and reduced emotional volatility. Others effectively learned and applied the two CBT-based skills that constitute the self-determination core value in the curriculum – deep breathing and positive thought replacement – improving emotional regulation: *“It really helped me think about how to… remove the anger and replace it with a positive thought… I used it when I was in my class… I was angry*,* but I just thought about no detention… so I just was quiet.”* (Scholar).

Accountability also resonated strongly with many case study scholars, a view supported by their school staff, parents and counsellors. Through action and reflection, they explored and internalised BAM’s archetype of ‘manhood,’ forming a personal connection to the ideals it represented. This framework guided their aspirations to become mature, respected young men—men who take responsibility for their actions and treat others with the same respect they seek for themselves.

Counsellors’ authenticity, the consistency of their presence and their willingness to share their own experiences helped to bring this abstract archetype and the other core values to life. It also helped to create what staff described as a “safe container” for action and reflection. This safe space was reinforced by the counsellors’ ability to demonstrate unconditional positive regard, model pro-social values and sustain a supportive group environment grounded in trust and mutual understanding. Case study scholars and their parents consistently described counsellors as people who had lived through similar challenges, including similar racialised or socio-economic experiences, making them more relatable and credible.

These shared experiences helped counsellors foster conditions in which scholars could engage with BAM’s group conditions— have fun, be safe and challenge yourself and others to be open and honest. Case study scholars who engaged in action and reflection did so because they adhered sufficiently to these three conditions. They could then share freely and experiment with new skills and identities. These scholars often had a pre-existing motive that drove them to commit to the conditions. Some were searching for a role model to guide them. Others were looking for support in managing anxiety, boosting self-esteem or understanding and addressing the impact of racism in their lives. For some, BAM’s influence filled gaps left by limited external support. For others, BAM and their wider support system reinforced one another.

Convergent data from the 11 case studies indicated that three key factors limited BAM’s impact for some of them. First, implementation challenges at Birch, where scholars attended only four sessions on average in year two, hindered engagement. Second, strong pre-existing support networks sometimes reduced BAM’s added value, especially among Tier 1 scholars with fewer needs. Third, some scholars’ developmental trajectories were influenced by factors beyond BAM’s control. Most case study scholars ranked (in no particular order) family, friends, faith or football as more important sources of support than BAM. For some, their increased integrity and accountability were attributed to natural development and growing maturity. Others faced greater emotional volatility during adolescence, which created more uncertain developmental paths. Scholars with SEND encountered additional challenges in aligning with the core values. In cases where extra support was needed but unavailable, scholars’ well-being was negatively affected, limiting BAM’s overall impact.

### RQ3: The Extent to Which Improvements in Social-Emotional Development Contributed to Improved Academic Engagement and Reduced Involvement in Crime/Anti-social Behaviour

Convergent evidence from counsellors, parents and most case study scholars indicated that BAM’s core values empowered scholars to make more responsible decisions. Many were motivated by their developing sense of integrity and accountability to make positive choices. Some applied themselves more at school, while others improved relationships at home and with peers by being more honest, understanding their emotions better and developing self-confidence.

Many case study scholars felt that BAM helped them handle situations more calmly, such as provocations from peers or teachers or encounters with the police. This ability to stay composed, make more responsible decisions and remain aligned with BAM’s core values contributed to fewer detentions and reduced conflict: *“I started with anxiety*,* like*,* coming to a new school*,* new friends … they could peer pressure you to do something that you don’t want to do at all*,* but then when I joined BAM*,* I felt welcomed … It just made me make the right choices”* (Scholar).

For some case study scholars, BAM’s core values were reinforced by support from family, mentors or other services, motivating them to act responsibly. Ambitions to be role models or guidance from other mentors strengthened their commitment to the values. However, external factors such as unstable school environments (limited resources, high staff turnover, staff absences), difficult parental relationships or financial stress weakened BAM’s impact for others. These scholars struggled to engage fully, as unresolved issues distracted them and made it harder to apply the core values consistently in their daily lives.

### RQ4: The Extent to Which Implementation Contributed to Unintended Consequences

Based largely on YG’s insights into the most frequent unintended consequences during 20 years of delivering BAM in the US, we were particularly alert to the impacts on school engagement and performance of negative group labelling, fractious BAM groups and time out of lessons. Starting with the issue of negative group labelling, unbalanced group composition (more Tier 3 scholars than the indicative target split of 15/70/15 according to the HSA) risked reinforcing BAM as a programme for “bad students”. This concern emerged during the feasibility phase due to recruitment challenges but was mitigated during the pilot phase through improved recruitment practices. Counsellors and school staff at Oak and Pine ensured that groups were not weighted towards students regarded widely among their peers and teachers as being most associated with poor behaviour. At Birch, BAM’s positive reputation from the feasibility phase prevented negative perceptions. All scholars and counsellors who spoke on the matter regarded BAM as prestigious.

Group factions emerged but were not clustered according to whether they were regarded as badly behaved in the wider school. According to counsellors, rather than leading to irresponsible decision-making, these cliques impeded implementation by challenging counsellors’ authority, undermining the group conditions and slowing down progress through the curriculum.

No counsellors, scholars, or parents reported negative academic impacts from missing lessons. Counsellors mitigated any impacts through rotating the lesson during which BAM was held, avoiding scheduling clashes with core subjects, and holding ‘academic integrity’ check-ins to track schoolwork and grades. However, some school staff expressed concerns, noting that frequent school-hour interventions could affect learning, particularly when a student was engaging in multiple interventions during school hours. Nonetheless, they acknowledged the social-emotional and behavioural benefits BAM provided.

## Discussion

There is insufficient evidence of what works to prevent youth violence in the UK, including gender-transformative programmes for boys. Several US-based interventions targeting youth development, education and crime reduction have struggled to replicate their positive effects in the UK and Europe more widely. This is arguably due in part to differences in service systems, cultural norms and school environments, which complicate implementation (e.g., Skärstrand et al., 2014; Baldus et al., 2016; Humayun et al., 2017; Fonagy et al., 2018; Segrott et al., 2022). Flawed programme adaptation efforts can also unintentionally undermine their ToC, reducing effectiveness (Movsisyan et al., 2019). Recognising these challenges, BAM was carefully adapted (Green et al., 2023b) before being evaluated in the UK. A theory-informed method was used to address the criticism that evaluations often focus insufficiently on the causal logic of interventions (Breuer et al., 2016).

Findings from the evaluation were mixed. While the target for mean sessions attended was met, group size, reach and curriculum progression fell short due to MHF’s resource limits, school-level challenges and counsellor competency gaps. However, evidence indicated that BAM contributed positively to case study scholars’ social-emotional development and responsible decision-making. Counsellors, parents and school staff reported instances of improved behaviour and emotional regulation among case study scholars who engaged consistently with the programme. The ToC clarified how the programme operates, for whom, under what circumstances, and why. This framework provides a valuable foundation for future adaptations and evaluations in new contexts.

Given the complexity of conducting a large-scale evaluation, there are important considerations for future research. While an RCT could provide robust evidence on BAM’s effectiveness in the UK, such a study could require significant expansion of MHF’s delivery infrastructure, potentially risking implementation quality. In terms of evaluation design, several key challenges emerged, particularly around the administration of outcome measures (Green et al., 2024). Given this, and evidence that BAM contributes to positive outcomes when delivered well, future evaluations of BAM might instead consider addressing implementation challenges and testing the effectiveness of these efforts.

The wider significance of this study is three-fold. First, it demonstrates how a successful US-based intervention can be adapted and implemented in a new context. Adaptation occurred through a twin-track process during the pre-implementation phase (Green et al., 2023b). Adjustments were made to implementation support to enhance intervention-context fit, informed by the ToC and key contextual factors (Craig et al., 2018; Damschroder et al., 2022) – for example, revising the school-level agreement between MHF and participating schools to use more collaborative and accessible language, in recognition of BAM’s lower profile in the UK compared to the US. Curriculum adaptations included both “surface” and “deep” changes. Surface changes involved tailoring language and cultural references to the London context – for instance, replacing “city blocks” with “streets,” basketball with football examples, and US films with those more familiar to UK youth. Deep adaptations affected core elements of the ToC. For example, the original archetypes of ‘savage’ and ‘warrior’ used to distinguish destructive and constructive expressions of anger were replaced with the terms ‘destructive’ and ‘constructive’, in response to concerns about violent connotations and colonial associations. The adaptation process was co-led by a diverse team, including YG, MHF, Black Thrive and BAM counsellors.

Second, the evaluation strengthens the evidence base on preventing youth violence in the UK by identifying the key factors that enabled BAM’s successes and the challenges that constrained it. BAM’s relatively high cost supported a comprehensive implementation package, including well-trained counsellors embedded in schools multiple days per week, fostering strong relationships with staff and scholars. These benefits were reinforced by BAM’s strong ToC, iterated over two decades and grounded in evidence, scientific theory, practitioner insight and scholars’ experiences. Two challenges are worth highlighting. School context was critical, requiring not only initial alignment with programme goals but also ongoing, well-resourced relationship management. Research on implementation in schools consistently shows the importance of a dynamic process in which those involved unite on what is being implemented, reflect and adapt in real time to address challenges and attend to contextual factors – notably structures and systems such as time allocation (Moore et al., 2024). Issues encountered around withdrawing students from lessons to attend BAM and a perceived misalignment in ethos between BAM and the wider school could arguably have been addressed had this approach been more fully adopted. However, a critical limitation was the lack of full buy-in from school leadership at the outset. As MHF had limited choice in initial school partnerships, this constrained the ability to negotiate and secure agreement on core elements of programme delivery. Without this foundational alignment, leverage to adapt or resolve implementation challenges was significantly reduced once delivery had begun. Additionally, scaling placed strain on BAM, as expansion during the pilot stretched MHF’s capacity to provide high-quality implementation support. These findings underscore the need for careful, deliberate approaches to scaling, ensuring that implementation quality is not compromised in the pursuit of expansion.

Third, the study makes a methodological contribution by combining realist evaluation and contribution analysis. This approach leverages the former’s sensitivity to context and mechanisms alongside the latter’s broader view of the intervention’s full causal pathway through a ToC. Contribution analysis also enhanced stakeholder engagement and transparency compared to realist evaluation alone.

This methodological innovation was a strength of this study, as was the inclusion of multiple stakeholders at different stages, ensuring rigour through the inclusion and triangulation of multiple perspectives. For example, the feasibility phase played a crucial role in refining the ToC and data collection methods, especially qualitative data from scholars. As for study limitations, the focus on BAM’s contribution to scholars’ development may have overshadowed other influences. Despite the use of open-ended questions, BAM’s prominence in the evaluation context likely shaped participants’ responses, potentially leading them to emphasise its impact more than other contributing factors. This particularly applies to interviews with young people, where prompts explicitly encouraged reflection on BAM-related influences on their journey during the two years, although in mitigation prompts also referenced other non-BAM influences. There is also a risk of bias in relying on counsellor-reported adherence, although it was only one aspect of implementation that was measured and the somewhat disappointing results in that respect suggest that counsellors did not paint an excessively positive picture. A final important limitation is that the study did not measure the impact of the programme on participants’ attitudes and behaviours towards women, while the non-implementation of the Respect for Womanhood content means that it is not possible to comment on its acceptability or contribution to outcomes.

## Conclusion

This pilot study suggests that BAM has the potential to support young people’s social-emotional development in the UK in ways that may reduce their involvement in violence. However, further adaptation and implementation support are needed to enhance feasibility and effectiveness. Future evaluations should focus on addressing implementation challenges and testing the effectiveness of these efforts, including on gender norms and attitudes towards GBV. A theory-driven approach that integrates contribution analysis and realist evaluation, as demonstrated in this study, could provide a rigorous framework for understanding not just *whether* interventions like BAM work, but *for whom*, and *under what circumstances* and *why*.

## Supplementary Information

Below is the link to the electronic supplementary material.

Supplementary material 1

## Footnotes

1
https://www.youth-guidance.org/bam-becoming-a-man/.

2 A senior member of the YG team overseeing the quality of BAM implementation.

3 Pseudonyms are used for the schools (Oak, Birch and Pine) and for the groups (Groups A through I).

4 General Certificate of Secondary Education exams are typically taken by students in Year 11 (ages 15–16 years).
