# Supplementary material for: Promoting Positive Youth Development with Adolescent Boys in UK Schools: A Theory-Driven Evaluation of the “Becoming a Man” Programme
Source: J Prev (2022). 2025 Dec 8;47(2):285–310. doi: 10.1007/s10935-025-00888-1 (PMC13046572; doi:10.1007/s10935-025-00888-1)
Supplement: Supplementary file 1 — Supplementary material 1 [file 10935_2025_888_MOESM1_ESM.pdf]

BAM
Theory of Change

This theory of change explains how BAM is expected to make a difference in young people's lives. By clarifying the model, guiding our decisions for the evaluation, and giving us a reference point from which to build, the theory of change will help us to deliver and study BAM.

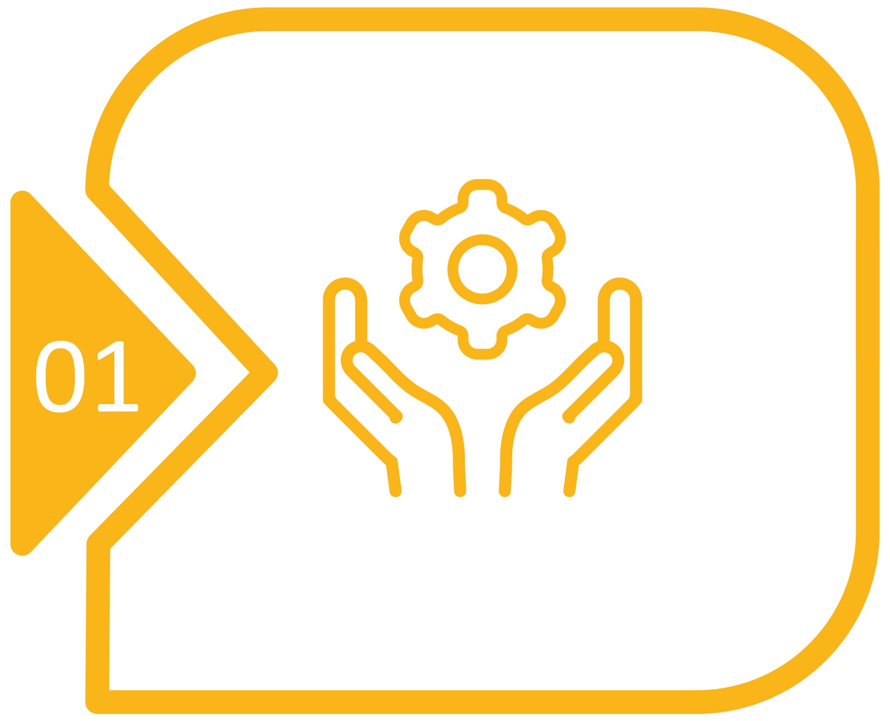

Activities

Counsellors deliver four activities with young people. The most important is the **BAM Circle**, a group of 8-to-12 members that meets once a week in school.

If counsellors receive high quality **backbone support**, this will create a **positive learning environment** to help them implement the BAM Circle and other activities successfully.

This will be influenced by how enabling the **school context** is and how engaged young people are in **action and reflection.**

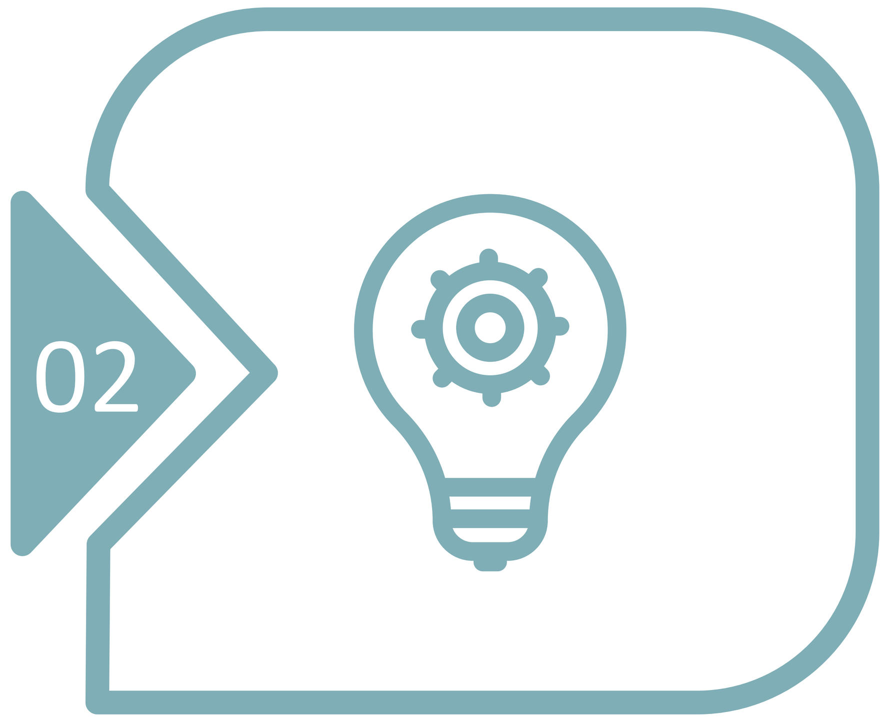

Intermediate Outcomes

These activities support young people to **internalise the six core values**: integrity, self-determination, positive anger expression, accountability, respect for womanhood, and visionary goal-setting.

They do this by helping them to: **actively** experience each value and **reflect** on these experiences.

This will be influenced by how ready and motivated young people are to **adhere to the group conditions.**

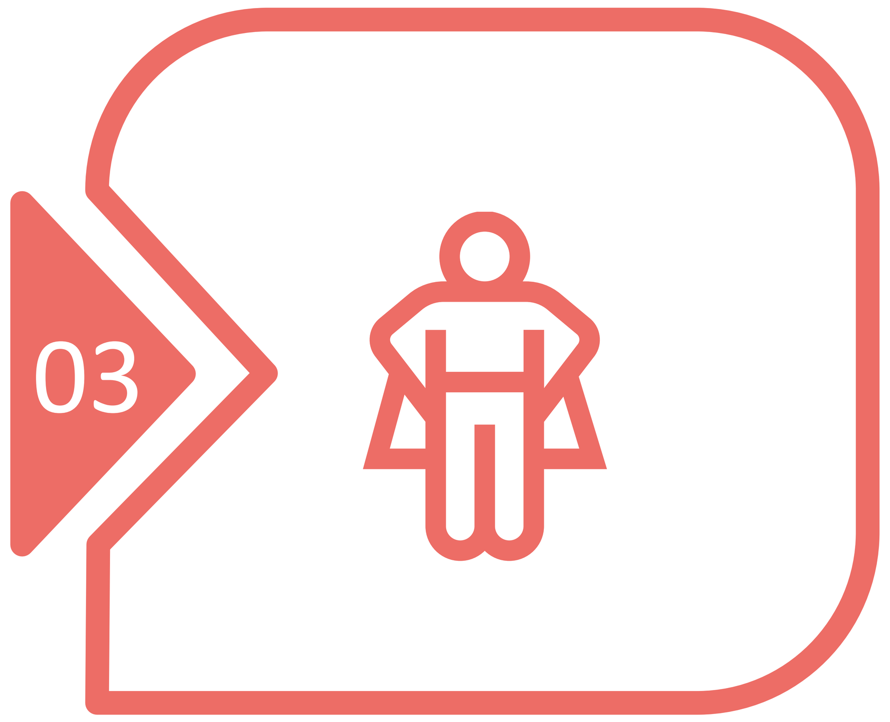

Ultimate Outcomes

These core values help young people to engage in **responsible decision-making**. This includes improvements in **educational attainment** and reduced involvement in **youth violence**.

They do this by **protecting** young people from risks and **empowering** them to capitalise on opportunities.

This will be influenced by the wider **social determinants of health** in young people's lives.

Activities

If counsellors receive high quality support from the 'backbone team', then this will help them to implement BAM successfully. This is because counsellors will feel their development is being nurtured in a positive learning environment. This will be influenced by how enabling the school context is and how engaged young people are in action and reflection.

1A

Backbone support

Effective recruitment and ongoing training and coaching support counsellors to deliver the curriculum and develop their competencies. This builds on the foundations put in place by the ManKind Project's New Warrior Training Adventure. They receive project management support and peer supervision from MHF, who must have a proactive and supportive working culture that is aligned with BAM's core values.

The advisory council help to liaise with communities and stakeholders alongside Black Thrive and Colourful Minds, who also help counsellors to apply an anti-racist ethos and respond to participants' mental health needs

School context

An enabling school culture and supportive staff help to create an environment in which counsellors feel able to deliver BAM well. This depends on:

- Whether counsellors show 'systemic leadership', which involves actively nurturing relationships with school staff.
- The school implementation team. This is a formal, problem-solving partnership between school leadership and BAM.
- Support from Colourful Minds to help schools respond to young people's mental health needs.

Engagement in action and reflection

Young people that engage more in action and reflection, which drives internalisation of the core values, are more likely to attend activities. They also make it easier for counsellors to deliver with quality and fidelity.

This creates a reinforcing feedback loop: good delivery supports engagement, which supports better delivery.

1B

Positive learning environment

Given these conditions, backbone support helps counsellors to feel their development is being nurtured within a positive learning environment. This means they feel that:

- They are essential and knowledgeable partners
- They are psychologically safe to try new methods
- There is sufficient time and space for reflective thinking
- Leaders express their own fallibility and need for counsellors' input

1C

Successful implementation

A positive learning environment helps counsellors to implement BAM successfully:

- Adaptation: Counsellors adapt activities to London while retaining their underlying function.
- Recruitment: Participants present with social-emotional challenges on entry but have different levels of need to prevent negative labelling.
- Quality: Counsellors demonstrate effective clinical work, group work, modelling, youth engagement and men's work (their own internalisation of the core values).
- Fidelity and attendance: Activities are delivered as intended to enough young people.

Intermediate Outcomes

If counsellors **implement BAM successfully**, then this will help young people to **internalise the core values**. This is because of the way in which young people will **actively experience** each value before **reflecting** on their experiences. This will be influenced by how ready and motivated young people are to **adhere to the group conditions.**

1C Successful implementation

A positive learning environment helps counsellors to implement BAM successfully:

- **Adaptation:** Counsellors adapt activities to London while retaining their underlying function.
- **Recruitment:** Participants present with social-emotional challenges on entry but have different levels of need to prevent negative labelling.
- **Quality:** Counsellors demonstrate effective clinical work, group work, modelling, youth engagement and men's work (their own internalisation of the core values).
- **Fidelity** and **attendance:** Activities are delivered as intended to enough young people.

Group conditions

Young people in BAM circles must learn to follow three rules: 'Have **fun**, be **safe** and **respectful**, and **challenge** yourself and others to be open and honest'. Only then can consistent, trusting relationships develop that create the foundations for **action and reflection**.

To follow these rules, young people must be sufficiently **ready**. This is influenced by their developmental stage, and whether their relationships (e.g. at home) and experiences (e.g. of trauma or of other services) have equipped them with the ability to form close bonds with others.

They must also be **motivated** to follow the three conditions. This depends on:

- Their fear of failure or humiliation
- Whether being open, vulnerable and respectful aligns with their preferred image of themselves, or the expectations and pressures placed on them by others
- Pre-existing relationships with others in group

2A Action and reflection

Successful implementation, given adherence to the group conditions, helps young people to engage in action and reflection. There are three **action** processes:

- **Experiential learning:** Young people learn actively, not passively.
- **Archetypes:** They receive clear models of behaviour to aim for.
- **Goals:** The activities help them to set, track, and celebrate goals.

During **reflection**, young people internalise the core values through Irvin Yalom's 11 **therapeutic factors**. These capture how **'here-and-now'** learning facilitates hope, emotional expression, relational awareness, and social learning.

2B Internalise the core values

Through action and reflection in the context of consistent, trusting relationships with the counsellor and group, young people experience **social-emotional and identity development**.

This happens through **internalising the six core values**: integrity, self-determination, positive anger expression, accountability, respect for womanhood, and visionary goal-setting.

Integrity is the first value, and the one upon which all the others build: young people know what kind of men they want to be, and can articulate and act in alignment with this vision of themselves.

Ultimate Outcomes

If young people internalise the core values then this will help them to engage in responsible decision-making, including improved educational attainment and reduced involvement in youth violence. This is because the core values will help to protect and empower them. This will be influenced by the wider social determinants of health influences in young people's lives.

2B

Internalise the core values

Through action and reflection in the context of consistent, trusting relationships with the counsellor and group, young people experience social-emotional and identity development.

This happens through internalising the six core values: integrity, self-determination, positive anger expression, accountability, respect for womanhood, and visionary goal-setting.

Integrity is the first value, and the one upon which all the others build: young people know what kind of men they want to be, and can articulate and act in alignment with this vision of themselves.

Wider influences

Wider social determinants of health in young people's lives influence how much the core values protect and empower them, through their influence on:

- The expectations young people perceive others to have of them.
- Pressure to conform to pro-social or anti-social norms.
- Relationships with positive or negative role models.
- Exposure to positive or negative developmental experiences.

Stronger, more positive influences reinforce young people's efforts to apply the core values. Stronger, more negative influences may limit their efforts.

These influences include other services, such as the community partners, Black Thrive and Colourful Minds, who will advocate for young people in the wider system, deliver mental health interventions to young people, and support interactions with partner services.

3A

Protecting and empowering

The core values act as coping mechanisms and positive assets, helping to:

- Protect young people against risks in their environment that might facilitate involvement in anti-social behaviour, including violence.
- Empower young people to thrive, by taking advantage of opportunities and resources to develop these assets further. This includes BAM and other interests and activities, as well as relationships with family, friends, and professionals.

3B

Responsible decision-making

By protecting and empowering them, the core values help young people to engage in responsible decision-making. This concerns the ability to make caring and constructive choices about personal behaviour and social interactions across diverse situations.

These choices help young people to stay in alignment with the core values and their sense of who they are. They also contribute to:

- Improvements in educational attainment
- Reduced involvement in youth violence

Unintended Consequences

If young people feel that BAM is for ‘bad kids’, then this will motivate them to engage in **irresponsible decision-making**. This is because **negative labelling** and **fractious BAM groups** will motivate young people to engage in anti-social behaviour. This will be influenced by whether **wider influences** in young people’s lives reinforce or limit these labels and hierarchies.

4A

Recruitment challenges

Young people develop a perception that BAM is for **‘bad kids’**. This message could be delivered through:

- Conversations with **counsellors**, **teachers**, **family** and/or **peers**
- **Communications** materials shared by MHF about BAM
- **Evaluation activities**, including surveys, which ask targeted, deficit-based questions
- The **weighting of BAM groups** towards young people with behavioural challenges.

BAM groups contain **factions** of young people with clear social hierarchies

Wider influences

- Participants are already battling **negative expectations** in other relationships with family/peers/school/ wider community
- Participants **lack relationships** that will help them to reframe their selection as something positive (e.g. an opportunity).

4B

Labelling and negotiating

- Recruitment for BAM may be experienced as **negative labelling** resulting in rejection of the school.
- The label of 'bad kid' may serve as a powerful form of **intervention capital**, being employed to enhance students’ status amongst peers.
- Groups containing factions may lead to the construction of **group ‘outsiders’** as students seek safety by strengthening pre-existing relationships.
- Students may seek to **renegotiate positioning within group hierarchies** by ‘bragging’ about and reinforcing anti-school activities, supporting further engagement in anti-social behaviour.

4C

Irresponsible decision-making

Both negative labelling and internal negotiation of positions within BAM Group hierarchies motivate young people to engage in **irresponsible decision-making**, including disengagement from school, while undermining their motivation to engage in pro-social behaviour.

Additional File 1: CONSORT checklist

CONSORT 2010 checklist of information to include when reporting a pilot or feasibility trial*

Section/Topic	Item No	Checklist item	Reported on page No
**Title and abstract**			
	1a	Identification as a pilot or feasibility randomised trial in the title	1
	1b	Structured summary of pilot trial design, methods, results, and conclusions (for specific guidance see CONSORT abstract extension for pilot trials)	1
**Introduction**			
Background and objectives	2a	Scientific background and explanation of rationale for future definitive trial, and reasons for randomised pilot trial	1-2
	2b	Specific objectives or research questions for pilot trial	2-3
**Methods**			
Trial design	3a	Description of pilot trial design (such as parallel, factorial) including allocation ratio	5-10
	3b	Important changes to methods after pilot trial commencement (such as eligibility criteria), with reasons	8
Participants	4a	Eligibility criteria for participants	2-3
	4b	Settings and locations where the data were collected	2-3
	4c	How participants were identified and consented	4-5
Interventions	5	The interventions for each group with sufficient details to allow replication, including how and when they were actually administered	2-3
Outcomes	6a	Completely defined prespecified assessments or measurements to address each pilot trial objective specified in 2b, including how and when they were assessed	7
	6b	Any changes to pilot trial assessments or measurements after the pilot trial commenced, with reasons	n/a
	6c	If applicable, prespecified criteria used to judge whether, or how, to proceed with future definitive trial	5
Sample size	7a	Rationale for numbers in the pilot trial	5-6
	7b	When applicable, explanation of any interim analyses and stopping guidelines	n/a
**Randomisation**			
Sequence generation	8a	Method used to generate the random allocation sequence	n/a
	8b	Type of randomisation(s); details of any restriction (such as blocking and block size)	n/a
Allocation concealment mechanism	9	Mechanism used to implement the random allocation sequence (such as sequentially numbered containers), describing any steps taken to conceal the sequence until interventions were assigned	n/a

Additional File 1: CONSORT checklist

Implementation	10	Who generated the random allocation sequence, who enrolled participants, and who assigned participants to interventions	n/a
Blinding	11a	If done, who was blinded after assignment to interventions (for example, participants, care providers, those assessing outcomes) and how	n/a
	11b	If relevant, description of the similarity of interventions	n/a
Statistical methods	12	Methods used to address each pilot trial objective whether qualitative or quantitative	7-8
**Results**			
Participant flow (a diagram is strongly recommended)	13a	For each group, the numbers of participants who were approached and/or assessed for eligibility, randomly assigned, received intended treatment, and were assessed for each objective	12
	13b	For each group, losses and exclusions after randomisation, together with reasons	n/a
Recruitment	14a	Dates defining the periods of recruitment and follow-up	12
	14b	Why the pilot trial ended or was stopped	n/a
Baseline data	15	A table showing baseline demographic and clinical characteristics for each group	Tables 2 and 3 and Figures 3 and 4
Numbers analysed	16	For each objective, number of participants (denominator) included in each analysis. If relevant, these numbers should be by randomised group	n/a
Outcomes and estimation	17	For each objective, results including expressions of uncertainty (such as 95% confidence interval) for any estimates. If relevant, these results should be by randomised group	n/a
Ancillary analyses	18	Results of any other analyses performed that could be used to inform the future definitive trial	n/a
Harms	19	All important harms or unintended effects in each group (for specific guidance see CONSORT for harms)	n/a
	19a	If relevant, other important unintended consequences	n/a
**Discussion**			
Limitations	20	Pilot trial limitations, addressing sources of potential bias and remaining uncertainty about feasibility	22
Generalisability	21	Generalisability (applicability) of pilot trial methods and findings to future definitive trial and other studies	21-24
Interpretation	22	Interpretation consistent with pilot trial objectives and findings, balancing potential benefits and harms, and considering other relevant evidence	22
	22a	Implications for progression from pilot to future definitive trial, including any proposed amendments	21-24
**Other information**			
Registration	23	Registration number for pilot trial and name of trial registry	n/a
Protocol	24	Where the pilot trial protocol can be accessed, if available	n/a
Funding	25	Sources of funding and other support (such as supply of drugs), role of funders	24

Additional File 1: CONSORT checklist

	26	Ethical approval or approval by research review committee, confirmed with reference number	24

Citation: Eldridge SM, Chan CL, Campbell MJ, Bond CM, Hopewell S, Thabane L, et al. CONSORT 2010 statement: extension to randomised pilot and feasibility trials. BMJ. 2016;355.
*We strongly recommend reading this statement in conjunction with the CONSORT 2010, extension to randomised pilot and feasibility trials, Explanation and Elaboration for important clarifications on all the items. If relevant, we also recommend reading CONSORT extensions for cluster randomised trials, non-inferiority and equivalence trials, non-pharmacological treatments, herbal interventions, and pragmatic trials. Additional extensions are forthcoming: for those and for up to date references relevant to this checklist, see www.consort-statement.org.

The TIDieR (Template for Intervention Description and Replication) Checklist*:

Information to include when describing an intervention and the location of the information

Item number	Item	Where located ** Primary paper (page or appendix number)	Other † (details)
1.	**BRIEF NAME**<br>Becoming A Man (BAM)	______________	________________
2.	**WHY**<br>BAM's theory of change is centred on supporting participants ('scholars') to make responsible decisions, including improved educational attainment and reduced involvement in youth violence. This is achieved by helping scholars internalise six core values—integrity, self-determination, positive anger expression, accountability, respect for womanhood, and visionary goal-setting. These values act as protective factors, helping to buffer scholars against risk while empowering them to seize opportunities. BAM fosters this internalisation through experiential learning, where scholars engage in activities that embody these values and reflect on their experiences in group settings. Successful implementation depends on scholars' readiness to create and sustain healthy and open group environments.	______________	_______________
3.	**WHAT**<br>**Materials**: The BAM intervention relies on a structured curriculum for the group sessions known as the "BAMual," which contains 30 lessons designed to help scholars engage with the programme's core values. Each lesson includes detailed guidance for counsellors, covering key concepts, group missions, role-play scenarios, and reflective discussions. Scholars participate in activities that	______________	_______________

incorporate physical materials such as writing tools and sports equipment. Counsellors receive training materials, including lesson plans, video demonstrations, implementation checklists, and professional development plans. These resources are available through Youth Guidance (YG), the intervention developer.

4. **Procedures**: BAM comprises four key activities: ______________ ______________

- BAM Circles: Group sessions delivered within schools to groups of 8–12 scholars, replacing a lesson. Activities include role plays, group missions, check-ins, check-outs, and homework.
- Special Activities: Group events outside school to reinforce lessons learned in BAM Circles through team-based tasks and social interaction.
- Brief Encounters: Informal check-ins between counsellors and scholars, typically lasting less than 15 minutes and occurring in communal school spaces like corridors or playgrounds. These interactions help maintain engagement and support ongoing development.
- One-to-One Support: Individualised support for scholars with higher levels of need. These sessions offer emotional support in a confidential space and reinforce learning from BAM Circles.

WHO PROVIDED

5. BAM counsellors are responsible for delivering the programme. They are recruited from the communities in which the scholars live, ensuring relatability through shared lived experiences. Counsellors are required to have QCF Level 6 qualifications and experience working with young people in therapeutic or mentoring roles. Counsellors receive 300 hours of training and ongoing coaching provided by MHF and YG, to support quality of delivery and fidelity to the programme model. ______________ ______________

HOW

6. All four activities are delivered in-person. Between January and March 2021, group sessions were delivered online due to COVID-19 restrictions. The BAMual was adapted for online delivery. ______________ ______________

WHERE

7. The BAM intervention took place in three primary types of settings within schools: ______________ ______________

- Classrooms: BAM Circles were conducted in dedicated classroom spaces, providing a structured, formal environment for group sessions.
- Communal School Spaces: Brief Encounters occurred in informal settings such as hallways, playgrounds, or lunch areas, allowing for spontaneous, short interactions to maintain engagement.
- Private Meeting Rooms: One-to-One Support sessions were held in private, confidential spaces within the school to ensure emotional support could be provided without interruption or concerns about privacy.

WHEN and HOW MUCH

8. ______________ ______________

- BAM Circles: Delivered weekly for one hour per session, replacing a school lesson. Each year involves 25 sessions, with each lesson in the BAMual taking one-to-three sessions to complete.
- Special Activities: Held intermittently throughout the school year
- Brief Encounters: Occurred as needed, typically lasting less than 15 minutes.
- One-to-One Support: Provided on an as-needed basis for scholars requiring additional emotional support.

TAILORING

9. Scholars with higher levels of need received personalised one-to-one support to ensure the intervention met their individual circumstances. These sessions varied in frequency and duration based on individual needs.

MODIFICATIONS

10.‡ The BAM intervention was adapted for the UK context to ensure cultural relevance and effective engagement with scholars. These adaptations were both surface and deep:

- Surface adaptations included changes to language, symbols, and cultural references to better align with UK youth culture (e.g., replacing references to American sports with UK equivalents like football).
- Deep adaptations involved adjustments to core curriculum elements that concerned key programme mechanisms.

These adaptations were made during the pre-implementation phase and were continuously revisited based on feedback from scholars, counsellors, and local stakeholders. For more information see: Green, F., Axford, N., Eastmond, N., Berry, V., Mannes, J., Allen, K., Callaghan, L. and Hobbs, T. (2023b). Transporting an evidence-based Program to a New Country: A Narrative Description and Analysis of Pre-implementation Adaptation. *Journal of Prevention* [online] 44(6).

HOW WELL

11. **Planned**: The replication specialist provided ongoing coaching to support curriculum fidelity, guiding counsellors through the BAMual's curriculum, offering feedback during training sessions, and conducting regular assessments of their competencies. Fidelity was evaluated using routinely collected programme data, gathered by MHF. This data tracked counsellors' progress through the BAMual, ensuring that they were delivering sessions as planned. Additionally, fidelity was assessed

	through coaching assessments conducted by the replication specialist, which evaluated counsellors' competencies in key areas such as clinical skills and youth engagement. Interviews with scholars, counsellors, the replication specialist, and parents further helped assess adherence to the intervention by capturing participant experiences and perceptions of delivery quality.		
**12.‡**	**Actual**: There is strong evidence that the implementation of BAM varied by implementation domain. While adaptation and the quality of delivery were generally successful, recruitment and exposure experienced mixed results with particular challenges observed at the PRU. Evidence on the quality of counsellors' adherence to the manual was positive, but their ability to progress through the curriculum was limited. There is some evidence that counsellors' previous skills and experience supported the delivery effort. However, there is strong evidence that 'backbone support' provided by MHF and YG made an important contribution to implementation by creating a positive learning environment for counsellors. The extent to which this environment enabled implementation was influenced by COVID-19, engagement from scholars, and school context, which was particularly incompatible at the PRU to the extent that BAM was withdrawn from the PRU after one year.	______________	______________

** **Authors** - use N/A if an item is not applicable for the intervention being described. **Reviewers** – use '?' if information about the element is not reported/not sufficiently reported.

† If the information is not provided in the primary paper, give details of where this information is available. This may include locations such as a published protocol or other published papers (provide citation details) or a website (provide the URL).

‡ If completing the TIDieR checklist for a protocol, these items are not relevant to the protocol and cannot be described until the study is complete.

* We strongly recommend using this checklist in conjunction with the TIDieR guide (see *BMJ* 2014;348:g1687) which contains an explanation and elaboration for each item.

* The focus of TIDieR is on reporting details of the intervention elements (and where relevant, comparison elements) of a study. Other elements and methodological features of studies are covered by other reporting statements and checklists and have not been duplicated as part of the TIDieR checklist. When a **randomised trial** is being reported, the TIDieR checklist should be used in conjunction with the CONSORT statement (see www.consort-statement.org) as an extension of **Item 5 of the CONSORT 2010 Statement.** When a **clinical trial protocol** is being reported, the TIDieR checklist should be used in conjunction with the SPIRIT statement as an extension of **Item 11 of the SPIRIT 2013 Statement** (see www.spirit-statement.org). For alternate study designs, TIDieR can be used in conjunction with the appropriate checklist for that study design (see www.equator-network.org).

What is the data collection plan?
The data collection plan will act as a guide for the evaluation. It is a practical tool that operationalises our Theory of Change (ToC). Each item within each sub section of the ToC is specified in greater detail (where necessary), as are the associated methods, measures and practicalities for collecting and analysing data.

Field	Description
Description	Description of the item, copied and pasted from the ToC
ToC Component	The ToC component (drawn from Dalkin et al. 2015) + associated sub-section that each element sits within
Contribution claim element	One of the final stages of contribution analysis (which this evaluation draws from) is the construction of contribution claims, which summarise the degree to which some or all of the theory of change was verified, accounting for other key influencing factors. These contribution claims will be assembled at the level of ToC sub-sections (Activites, Intermediate Outcomes, Ultimate Outcomes, Unintended Consequences), by compiling evidence on the extent to which, in each sub-section:<br><br>•**Outcome happened**<br>• …due to MResP vs alternative mechs (based on evidence of extent to which **MResP happened**, and evidence on **link between MResP and Outcome**)<br>• …which was due to MResO (based on evidence of extent to which **MResO happened**, and evidence on **link between MResO and MResP**)<br>… the link between MResO and MResP was mediated by Context (based on evidence of extent to which **Context happened**, and evidence on **links between Context and MResP**)<br><br>The 'type of hypothesis' column identifies which part of the contribution claim for each sub-section that particular item is concerned with

Attributes	From Funnell and Rogers 2011: *Attributes relate to specific features of the outcome; for example, if changed farming practices are a desired outcome, what specific changes are sought? Is there a range of alternative acceptable behaviors? Typically attributes relate to quality, quantity, timeliness, cost, and priority target groups with which the outcome should be achieved* [this has been specified for priority elements (i.e. those upon which data will be collected from the start of deliver) where necessary, but not for others, either where the description is sufficient or where they are not a priority]
Pri/Sec/Ter method	Method used to collect data
Pri/Sec/Ter data collector	Stakeholder group responsible for collecting data
Pri/Sec/Ter research participant	Stakeholder group with whom data is being collected
Pri/Sec/Ter comparison/target	From Funnell and Rogers 2011: *Comparisons make it possible to tell whether the attribute is being achieved to a desired level, moving in the right direction, and so on. Standards, norms, targets, change in the desired direction over time, comparisons with baselines, and redistribution of outcomes among target groups are all examples of comparisons.*<br><br>Comparisons/targets can also refer to what we'd expect to see if the assumptions contained in the theory of change were borne out in reality
Pri/Sec/Ter TP1	Initial data collection timepoint for each element
Pri/Sec/Ter follow-up TP	Any and all follow-up timepoints (where applicable)
Notes	Additional notes

Values
Free text<br>(Activities;<br>Intermediate Outcomes;<br>Ultimate Outcomes;<br>Unintended Consequences)<br>+<br>(MResO (mechanism resource);<br>C (context);<br>MResP (mechanism response);<br>O (outcome))
Did Outcome happen;<br>Did MResP happen;<br>Did MResP lead to Outcome;<br>Did MResO happen;<br>Did MResO lead to MResP;<br>Did Context happen;<br>Did Context shape MResP

Free text
Free text
Free text
Free text

Free text
Free text
Free text
Free text

Item #	Description	ToC Component
17	Counsellors implement BAM successfully:<br><br>• **Recruitment**: Participants present with social-emotional challenges on entry but have different levels of need, to prevent negative labelling.	Activities - 1C (Outcome)

Contribution claim element

Did Outcome happen

Attributes

Young people and their parents/carers will be recruited to the intervention by the BAM counsellors and colleagues in the MHF. Participants are eligible if they are boys aged 12-14 years in one of three secondary schools in Lambeth and experiencing challenges in at least one area of their social-emotional development according to the HSA. However, all groups must incorporate a range of young people with different types and levels of need. Specifically, all groups and school cohorts will aim for approximately a 15%/70%/15% split across the three respective tiers of the HSA. This is to prevent negative labelling of participants. Counsellors will also work with school leadership to ensure that, as far as possible, groups are not made up of factions of young people. Each group will be made up of 8-12 young people. This is to ensure there are enough young people to participate in the activities and facilitate the development of group identity/belonging, but not too many young people such that the counsellor finds the group difficult to manage and the ownership that each young person over the space is diluted, making speaking up more daunting

Primary method	Primary data collector	Primary research participant
Routine data collection	Counsellors	Young people

Primary comparison/target	Primary TP1
Each counsellor is delivering five groups of 8 to 12 young people by end of 2021.	Jan-22

Primary follow-up TP	Secondary method	Secondary data collector
NA	Holistic Student Assessment (HSA)	Counsellors

Secondary research participant	Secondary comparison/target	Secondary TP1
Young people	All young people who take part in BAM are experiencing challenges in at least one area of their social-emotional development, according to the Holistic Student Assessment (HSA; Allen at al., 2017); All three tiers of need are represented in each group and in each school according to HSA, with a split of ~15%/70%/15% for tiers 1, 2 and 3 respectively	Jan-22

Secondary follow-up TP	Tertiary method	Tertiary data collector
NA	Qualitative interview	Evaluation Team

Tertiary research participant	Tertiary comparison/target	Tertiary TP1
Counsellors	Counsellors report being able to establish the intended size and balance of need in each of their groups, in their professional opinion. They report that they were able to create groups without any factions (i.e. schools were open to their requirements and supported them to set up groups with no factions, by sharing their insight on their cohort of students).	May-Jul 2023

Tertiary follow-up TP	Quaternary method	Quaternary data collector
NA	Qualitative interview	Evaluation Team

Quaternary research participant	Quaternary comparison/target
School staff	School staff report that counsellors were able to establish the intended size and balance of need in each of their groups, in their professional opinion. They report that they were able to create groups without any factions (i.e. schools were open to their requirements and supported them to set up groups with no factions, by sharing their insight on their cohort of students). They understand why creating diverse groups is important, and how and for whom BAM is supposed to be targeted at (i.e. YP experiencing challenges with social emotional development, not just YP with behavioural problems).

BAM Scholars: Topic Guide

Consent (5 mins)

- *Hand out info/consent sheet and talk through it*
- *Once they've signed, put the form to one side to come back to after the interview.*

Thank-you for agreeing to speak to me today. We'd like to invite you to help us to figure out whether and how BAM and your counsellor have helped you. To help us do that, we want to put BAM to one side to start with. We want to know what changes you've noticed in yourself, if any, over the last couple of school years, including both things you've found difficult as well as ways in which you've grown and developed. We'll then come back to BAM, to figure out if it has played a role in those changes.

Exercise: Hot Air Balloon (20 mins)

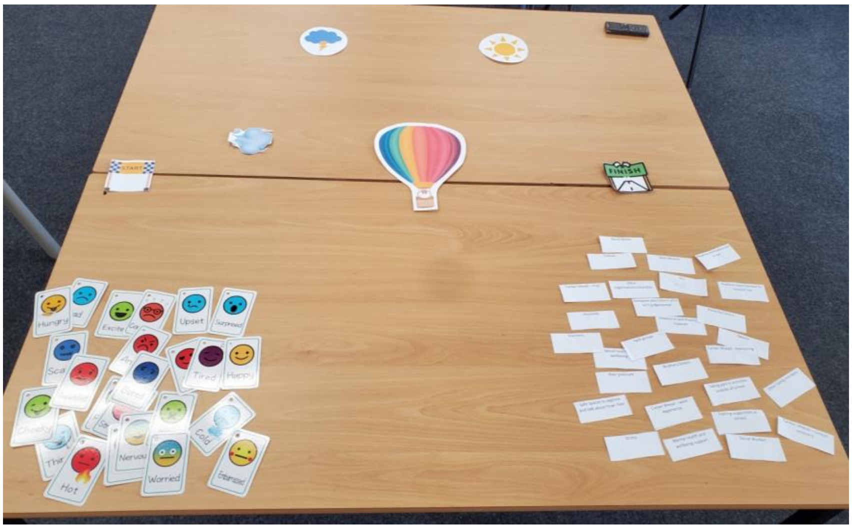

Resources:

- ***Hot air balloon" components:** start line, finish line, balloon w/ basket, wind, rain, sun*
- ***Feelings cards (bottom left):** Used during start/finish line components*
- ***Prompts (bottom right)**: Used during wind, basket, rain and sun components. These are prompts for young people to help them think about influences on their journey over the last two years, including:*
    - *BAM-related influences (the counsellor, the BAM Circle, brief encounters, one-to-ones, special activities)*
    - *Relationships (friends, parents, carers, brothers/sisters, cousins, aunties/uncles, grandparents, faith groups, teachers, social worker)*

- *Other factors (racism, negative/positive expectations, other services, role models, peer pressure)*

Introduction

We're going to invite you to think about your time over the last couple of years as a hot air balloon journey. Starting from September 2021 and running through to today, we're going to think about how you were feeling then, how you're feeling now, what's been the wind that's been driving you along, who's been riding in the basket alongside you, what's been the bad weather that's been getting in the way and the good weather that's made things easier.

Start/Finish Line

This section is focused both on changes in their social-emotional development (internalisation of the core values) AND changes in their behaviour (responsible decision-making)

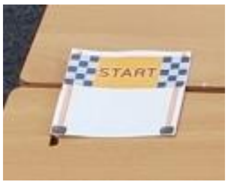

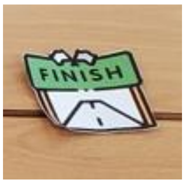

Let's start here, at the start line. Cast your mind back to a couple of years ago, September 2020, the start of Year 8/9/10 [will depend on interviewee]. We'd like to invite you to share how you were feeling about life back then, using some of these "feelings" cards.

- How were things at school?
  - Were you happy/sad/angry/confused? Why did you feel that way?
- How were things at home?
  - Were you happy/sad/angry/confused? Why did you feel that way?
- How were things going with your friends?
  - Were you happy/sad/angry/confused? Why did you feel that way?

Now let's fast forward to today. We'd like to know how you're feeling about life now.

- How are things at school?
  - Are you happy/sad/angry/confused? Why do you feel that way?
- How are things at home?
  - Are you happy/sad/angry/confused? Why do you feel that way?
- How are things going with your friends?
  - Are you happy/sad/angry/confused? Why do you feel that way?

The Wind

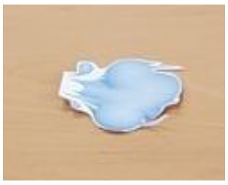

This section is focused on the extent to which BAM (vs alternative influences) was responsible for any change in (i) the core values, and whether that was due to action and reflection; and (ii) responsible decision-making, and whether that was due to the protective and empowering influence of the core values

[*Summarise their journey out loud, reading it back to check they're happy with your understanding*]. *Now, thinking back to the hot air balloon, we want to know what's been the wind for you over the last couple of years.*

If you had to pick a few things that have been pushing and driving you from where you were back then to where you are now, what would they be? Feel free to use these prompts, or to write your own, and put them under the "wind". These sources of "wind" could be people, experiences you've had, things that have happened or things you've seen.

- [*For each choice*] Why did you choose that?

Now, if you had to rank them from top to bottom, starting with the one that made the biggest difference, how would you order them? Why?

The Basket

This section is focused on context, as are the other weather sections - what things have been helping/hindering their journey, including people, experiences etc.

Commented [FG1]: This part is getting at context, as are the other weather parts. It's all about what things have been helping/hindering their journey, including people, experiences etc.

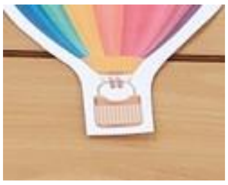

The second thing to think about with a hot air balloon ride is who's coming with you. These are the people who have been most closely involved on your journey this year, and supported you along the way.

So who's in the basket alongside you? Feel free to use these prompts, or to write your own, and put them under the "basket".

- [*For each choice*] Why did you choose them?

Now, if you had to rank them from top to bottom, starting with the one that made the biggest difference, how would you order them? Why?

Bad Weather

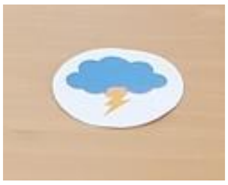

What are some of the things that have made the journey harder for you? Feel free to use these prompts, or to write your own, and put them under the "raincloud".

Now, if you had to rank them from top to bottom, starting with the one that made the biggest difference, how would you order them? Why?

Good Weather

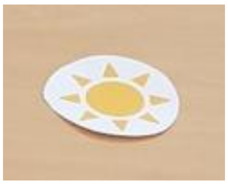

What are some of the things that have made the journey easier for you? Feel free to use these prompts, or to write your own, and put them under the "sun".

Now, if you had to rank them from top to bottom, starting with the one that made the biggest difference, how would you order them? Why?

BAM (20 mins)

[NB for interviewers: for discussion around BAM and BAM activities, use prompts and follow-ups that help YP to articulate how these may have led to changes in how YP think and feel about themselves. This will help us to test the extent to which BAM activities function as intended. Specifically, the idea is that BAM circles offer young people the opportunity to actively experience both themselves and others being in and out of alignment with the core values, which supports their connection with and comprehension of those values. It then offers them the space to reflect on these experiences, by discussing them with others in the group, thereby engaging Yalom's group therapeutic factors and supporting internalisation of the core values. Other activities help to supplement/reinforce work done in the BAM circle]

General

[*If not already discussed*] What role has BAM played in your journey? How does it compare to the other influences you mentioned? Why is the contribution more/less?

Activities

[*Pull out the prompt cards for the four BAM activities – BAM Circle, brief encounters, one-to-ones, special activities*] If you had to rank these BAM activities from top to bottom, starting with the one that made the biggest difference, how would you order them?

- [*For each activity*] Why did you place it there? How did it help more/less than the other activities?
- [*If not already discussed*] What does your counsellor do well? What could they do differently?

Recruitment

This section is focused on potential unintended consequences of BAM. An important part of BAM is that scholars develop a sense of belonging to their group specifically and BAM more generally. Lots of things affect this, including recruitment – it is important that participants present with social-emotional challenges on entry but have different levels of need, to prevent negative labelling. So this question is to get at whether and how the recruitment process communicated to them that BAM was something to be proud of.

When did you first hear about BAM? How did you feel about it? What do other students at school think about it?

Attendance

Why did you keep going to BAM?

Improvements

Thank-you for taking the time to speak to me about BAM. We are interested in any ideas you have for changes or improvements that you think could be made to BAM…

- What do you think could be done to make BAM better?

AFTER INTERVIEW IS FINISHED

- *Take photos of the journey they've mapped out.*
- *Take their email and name for vouchers*
- *Taking a picture of the signed consent form and share it, then upload to sharepoint.*

BAM Counselor Core Competencies

Introduction to BAM Counselor Rubric

Include language here that describes the purpose of the rubric, the need for BAM counselors to self-assess and for the counselor and coach to agree on and prioritize an area for focus over a specified timeframe.

Competency	Gold Standard	Developmental	Unacceptable
**Clinical Processing**<br>Listening<br>Assessing<br>Observing<br>Empathizing<br>Paraphrasing<br>Summarizing<br>Clarifying<br>Reflecting<br>Reframing<br>Probing<br>Challenging<br>Confronting<br>Connecting	**Listening**<br>Uses listening to convey interest, understanding, positive regard, and genuine caring for each student. Listens for and hears the meaning under the words. Listening is utilized to enhance relationships with students and build the foundation for deeper, more challenging work.<br><br>**Here and Now**<br>Accurately assesses and consistently identifies teachable moments during the group. Knows how and when to shift from content to "here and now" processing in ways that keep students engaged and connects what is happening to the BAM Values. Can take any lesson, activity, story and consistently make connections between the "here and now" and students' lives outside of the circle. Routinely generates "aha" moments for students and helps students generalize their learning in the BAM circle to other areas of their lives.	**Listening**<br>Listens carefully to students but does not consistently convey understanding. Counselor uses a limited set of processing skills to catalyze deeper understanding and reflection.<br><br>**Here and Now**<br>Occasionally utilizes opportunities to make connections between what is happening in the group and the BAM Values.	**Listening**<br>Does not demonstrate effective listening skills. Primary mode is talking at students.<br><br>**Here and Now**<br>Moves through the curriculum, but rarely pauses to process what is happening in the group. Consistently misses opportunities for deeper engagement and learning. Does not debrief activities. Does not make connections between what is happening in the group and life outside of the circle.

	**Challenging and Confronting**<br>Consistently illuminates disconnects between stated values and behaviors. Tactfully confronts students' cognitive distortions by making observations and asking carefully crafted probing questions. Routinely challenges and "stretches" students while maintaining positive regard and safety of the individual and the group. Pushes students to engage in ruthless self-examination for the purpose of their growth and success. Manages own discomfort and "holds" students' discomfort throughout.	**Challenging and Confronting**<br>Inconsistently challenges and confronts students. Is not yet strategic in decisions about when, who, and how to confront. Some confrontations lead to breakthroughs for students, others do not appear to have intended result.	**Challenging and Confronting**<br>Does not point out or correct cognitive distortions. Does not create a culture of authenticity and challenge in the group.<br><br>OR<br><br>Confronts students in anger or is otherwise disrespectful to students. Group safety and trust is Compromised through inappropriate challenge and confrontation. Counselor not managing transference issues.
**Group Work**<br><br>It is interaction between group members that sets into motion the curative factors of the group, therefore, it is the counselor's task to create a group culture maximally conducive to effective group interaction. (Yalom)	Prioritizes the development of a safe container for group work in the early sessions and uses a wide range of tools to tend to the safety of the individuals in the group and the group as a whole throughout the program.<br><br>Effectively establishes group safety and cohesion and ensures group can hold whatever comes up in the group. Interpersonal conflict and strong emotions are permitted and supported within the group. Trust, regard, and group cohesion are strong enough to "hold" confrontation and challenge in the group.	Expectation of group safety is communicated, but not consistently reinforced. Some incidents of trust being violated still in evidence. Uses a limited set of tools and skills to foster group cohesion.<br><br>Utilizes a variety of tools to facilitate group safety and cohesion, however, interpersonal conflict and strong emotion is unevenly accepted and still presents a threat to group cohesion.	Does not prioritize group development and cohesion, safe container is not formed.<br><br>The group is unsafe and is not cohesive. There are incidents of physical violence, verbal abuse, and/or high levels of disengagement. BAM Counselor relies on "discipline" mode to redirect students.

	Routinely communicates, lectures on, teaches, and makes connections to the 3 BAM Expectations: safety/respect, fun, challenge and has clearly established these norms within the group.<br><br>Consistently and strategically utilizes the check-in process to maximize group cohesion and peer relationships and learning. Consistently facilitates group members helping one another; asks questions to draw group members out and has them share their stories and approaches to problems with one another.<br><br>Counselor has knowledge of Yalom's theory of group work and routinely facilitates and leverages group curative factors. Explicitly calls out and utilizes Yalom's curative factors to accelerate and deepen group work.<br><br>Consistently helps students generalize from youth engagement work to core values and larger goals of program. Makes observations and asks questions that routinely facilitate "aha" moments for students.<br><br>Demonstrates strong facilitation skills; asks probing questions and makes powerful observations that deepen individual and group learning.	Uses multiple strategies and modes to communicate and reinforce the 3 BAM Expectations: safety/respect, fun, challenge.<br><br>Effectively teaches, models, and prioritizes the importance of the check-in to establish and maintain group safety and cohesion. Checkins are utilized, however, not all students are checking in authentically. Group cohesion emerging, but not yet solidified.<br><br>Has knowledge of Yalom's curative factors and emerging skill in facilitating the experiences of the group to maximize curative factors. Occasionally leverages the experience of individual group members to facilitate learning in the group.<br><br>Misses some opportunities to make connections between youth engagement activities, the BAM Values, and the larger goals of the program.<br><br>Demonstrates basic facilitation skills and demonstrates emerging skill in asking questions.	Does not effectively communicate or inconsistently reinforces the 3 BAM Expectations:<br>safety/respect, fun, challenge.<br><br>Inconsistently utilizes check-in process. Allows group members to skip check-in and/or not take the check-in process seriously. There are insults and disrespect during check-ins. There is a pattern of group members using something shared in group against someone outside of the group. Safety is violated.<br><br>Does not possess a working understanding of Yalom's curative factors. Little evidence of utilizing the benefits of the group to leverage learning.<br><br>BAM counselor does not make many connections between group activities and BAM values. Prioritizes "getting through" the curriculum at the expense of helping students make important connections.<br><br>Does not demonstrate basic facilitation skills. Does not ask well formed questions or make accurate observations.

**Modeling**<br>Learning theory posits that people learn from one another, via observation, imitation and modeling: from observing others, one forms an idea of how new behaviors are performed and on later occasions this coded information serves as a guide for action. (Bandura)<br><br>The MKO (More Knowledgeable Other) refers to anyone who has a better understanding or a higher ability level than the learner, with respect to a particular task, process or concept. The MKO is normally thought of as being a teacher, coach, or older adult, but the MKO can also be peers or a younger person. (Vygotsky)	BAM Counselor is the tool for the youth learning the BAM values; he has internalized the values, uses them to guide his life and makes choices aligned with values. He makes frequent and strategic use of his own story, experiences, and choices to teach important lessons to students.<br><br>BAM Counselor demonstrates high level of self-awareness and acknowledges when he falls out of integrity.<br><br>Uses self-disclosure with the sole purpose of teaching, facilitating, catalyzing the group and consistently makes strong connections between his own personal narrative and the BAM Values. Shares aspects of his own story and journey becoming a man to inspire, instruct, and facilitate the therapeutic growth of the client.<br><br>Shows accountability and models ruthless self-examination and vulnerability with therapeutic appropriateness. Makes amends for mistakes or lapses and transparently	BAM Counselor has an emerging sense of “self” as a primary tool for catalyzing youth learning. Occasionally uses his own story, experiences, and choices to teach important lessons to students.<br><br>Demonstrates self-awareness, but still has several blind spots that trip him up.<br><br>Self-discloses in the group, but purpose is not always clear; does not always make strong connections back to the BAM Values. Emerging sense of one’s own story & voice and the importance of strategically sharing parts of own story to facilitate growth students.<br><br>Shows accountability, but does not yet strategically model self-examination in the group. Self-disclosure not always clearly tied to the purpose of student learning and	BAM Counselor does not check-in regularly in the BAM Circle and rarely uses his own story, experiences, and choices as text for teaching.<br><br>Evidence of low level of self-awareness.<br><br>BAM Counselor uses either no self-disclosure or too much self-disclosure. Rather than modeling, counselor is routinely “doing his own work” with inappropriate levels of personal sharing.<br><br>Counselor exhibits low accountability. Behavior is consistently disconnected from BAM values<br>e.g., comes to group late; is

	models "doing the work" of manhood.<br><br>Demonstrates confidence and skill in taking students on a journey of deep introspection through emotional presence, attunement, and probing questions.<br><br>Displays high level of emotional health and well-being and is present and accepting of strong emotion of students - (ie., student comes in angry) Is comfortable with discomfort in self and students. Models willingness to "go deep" and take risks.<br><br>Models BAM values outside of the circle; always "on" - 24/7 - how they engage with other people, how they handle conflict, etc.<br><br>Models leadership behaviors such as: initiative, advocacy, care of others, responsibility, problem solving, collaboration, etc.	group growth. Makes amends for some, but not all lapses in integrity and accountability in the group.<br><br>Demonstrates emerging competence in taking students on deep journeys of introspection. Uses is a limited set of tools and is not yet fluid moving from challenge to support.<br><br>Evidence of adequate emotional health and well-being. Increasing comfort with discomfort and beginning to model “going deep” with the group.<br><br>Takes increasing responsibility for self as “model” of BAM Values and shows increasing awareness of the importance of one’s behavior in and outside of the BAM Circle.<br><br>Beginning to see themselves as a leader (identity) and demonstrates some leadership behaviors.	defensiveness when confronted; affirms bravado, machisto, or patriarchal attitudes.<br><br>Does not prioritize facilitating students’ introspection. Pattern of a preacher mentality - dominates the group with lecture<br><br>Does not tend to the one’s own emotional health and well-being. Is uncomfortable to the point of being ineffective when students express strong emotions.<br><br>Personal values consistently override BAM Values and become the focus. Evidence of BAM Values being violated; pattern of sexism, homophobia, or other isms going unchecked in the group.<br><br>Does not see themselves as a leader and does not practice leadership behaviors. Has not yet internalized an awareness of or importance of one’s own story and “presence” as a leader to the role of BAM Counselor.

**Youth Engagement** A sentiment young people feel towards a particular person, activity, place, or outcome. Involving young people in the creation of their own destinies. Facilitating meaningful participation of youth with passion and opportunities for youth to take responsibility and leadership while working in partnership with caring adults who value, respect, and share power with them. (Pittman)	Comes in fully prepared with deep knowledge of curriculum, materials ready to go, and lesson activities and a plan A and plan B.<br><br>Exudes comfort, confidence, and high energy. Consistently conveys positive regard for the youth and communicates warmth and acceptance. Consistently performs, give directions, facilitates activities, and tells stories in a comfortable, confident, and energetic manner - youth are drawn in and connected.<br><br>Counselor strategically makes connections between youth engagement, clinical process, and Men's Work.<br><br>Regularly utilizes natural observation; and is able to accurately assess how engaged youth are - and what is needed to increase engagement<br><br>Possesses a wide range of skills and tools needed to increase engagement at different points in the group icebreakers, energizers, group	Has knowledge of planned lesson, but is not consistently prepared with contingencies to increase youth engagement when needed.<br><br>Inconsistent energy, charisma, and presence in the group.<br><br>Occasionally, but not consistently makes connections between youth engagement, clinical process, and Men's Work.<br><br>Does not always seem to be "in tune" with the energy of the group and the level of youth engagement.<br><br>Utilizes a limited number of tools and approaches to energize the group and increase youth engagement with varying degrees	Counselor is unprepared to facilitate the lesson; does not know what is coming next, does not have materials ready, and/or does not have a plan to increase youth engagement when needed.<br><br>Pattern of low energy within the group. Consistently stoic and flat performance by counselor<br><br>Rarely makes connections between youth engagement activities, clinical process, and Men's Work. Counselor overemphasizes youth engagement without connecting it to process or men's work.<br><br>Counselor makes inaccurate assessments of group or makes judgments that are punitive (not clinically appropriate)<br><br>Counselor does not utilize youth engagement strategies; behaves like a disciplinarian, consistently

	missions, internal incentives, etc. Maximizes student engagement while effectively balancing fun, safety/respect, and challenge in the group.<br><br>BAM Counselor authentically and idiosyncratically uses their own personal style and story, while maintaining fidelity to the program, to build relationships with and engage the youth in the group.<br><br>I know who I am as a man and BAM Counselor and I know how to use who I am to meaningfully engage each of the group members.<br><br>High % of students are engaged a high % of time	of effectiveness. Youth engagement occasionally comes at the expense of safety/respect or challenge in the group.<br><br>Emerging sense of personal style and balancing of personal charisma, rapport building, and facilitation of the curriculum.<br><br>I am learning more about who I am as a man and BAM Counselor and I am beginning to use who I am to engage group members.<br><br>X% of students are engaged X% of the time. (An acceptable number, but room for improvement.)	shuts youth down and silences them. Only the BAM expectation of "fun" is emphasized, at the expense of safety/respect, challenge<br><br>Limited evidence of personal style or ownership of the curriculum. Delivery of content consistently falls flat. Or, personal charisma and style supercedes the curriculum and compromises student learning of the BAM Values.<br><br>I am uncertain who I am as a man or as a BAM Counselor and unclear about how to use who I am to effectively engage group members.<br><br>Only X% of students are engaged X% of time. (Unacceptable.)
**Systems Change Leadership**<br>Systems change leaders have a commitment to the health of the whole that radiates to nurture similar commitment in others. They are able to see reality through eyes of people very different from	BAM Counselor builds systemic relationships with family/parents, school staff, community members and partners in service of BAM students and the BAM program mission and goals. Has cultivated program champions at the school and in the community that believe in and support the program; has a "following" at the school. School staff are aware of and informed about all aspects of BAM Program. Is able to effectively advocate	BAM Counselor has built relationships with several key school staff and is in regular communication with staff, but has not yet cultivated sufficient BAM champions at the school and has not yet utilized the potential of partnerships in service of BAM student support.<br><br>BAM Counselor spends some time outside of the BAM room, but is still	BAM Counselor is out of relationship with school staff and administrators and/or is in conflict with staff and/or caught up in school politics. School staff are uninformed about the program; no real champions for the program at the school. Evidence of poor communications with school staff.<br><br>BAM Counselor spends little or no time outside of the BAM room and

themselves which encourages others to be more open as well. They build relationships based on deep listening, trust and collaboration. Systems leaders foster collective leadership through three capabilities: the ability to "see" the larger system, foster reflection and more generative conversations, and shifting collective focus from reactive problem solving to co-creating the future. (Senge, Kania, Hamilton)	for youth because of relationships with administrators and teachers. Respectfully navigates relationships with teachers even when teachers are "down" on students<br><br>BAM Counselor frequently and strategically spends time throughout the school community, in the hallways, classrooms, yard, etc. in service of assessment and relationship building.<br><br>Regularly attends school team meetings such as CARE team, IEP meetings, and leadership meetings as appropriate to advocate for students and bring clinical expertise to school wide challenges. Makes effective use of one-on-one mentoring and counseling of BAM youth between BAM meetings and knows students in their contexts.	not as present as necessary within the school community to effectively assess and build relationships.<br><br>Advocates for students in select instances, but does not yet take leadership for systems change work on behalf of BAM youth. Meets with students in crisis, but does not yet have a system in place to ensure knowledge of and support for every student between sessions.	has little or no knowledge of youth outside of what is shared in the group<br><br>There is little follow-up with or advocacy for youth between BAM meetings and little to no individual counseling.<br><br>BAM Counselor does not demonstrate awareness of systems barriers and/or does not communicate interest in taking leadership for systems change.<br><br>BAM Counselor spends little or no time outside of the BAM room and has little or no knowledge of youth outside of what is shared in the group

	Takes a systemic view and approach to the work; advocates for and facilitates positive change in the system on behalf of BAM youth. Is viewed as a leader and well positioned to influence positive change at the school. Knows how to navigate system obstacles and barriers and brings an action oriented, problem-solving approach .<br><br>Develops and utilizes effective communication systems with BAM youth, school staff and families.<br><br>Is always a strong and compelling spokesperson for BAM Program. Effectively engages funders and hosts and facilitates site visits that promote BAM; visitors leave visits inspired to support the BAM Program.<br><br>Develops and utilizes effective communication systems with BAM youth, school staff and families.<br><br>Is always a strong and compelling spokesperson for BAM Program. Effectively engages funders and hosts and facilitates site visits that promote BAM; visitors leave visits inspired to support the BAM Program.	Understands the need for the system to change to better meet the needs of students, but has not yet internalized or applied a systems change leader identity or approach. Has awareness of systems barriers in the school setting, but is not yet positioned to influence change. Tends to work more with teachers who express favorable views of BAM and BAM students.<br><br>Communicates with BAM youth, school staff, and families, but has not yet developed systems to sustain and ensure consistent communications on behalf of BAM student well –being.<br><br>Has an emerging sense of self as a spokesperson for BAM Program. Promotes the program when asked, but is not yet fully confident in role of host or spokesperson.	There is little follow-up with or advocacy for youth between BAM meetings and little to no individual counseling.<br><br>BAM Counselor does not demonstrate awareness of systems barriers and/or does not communicate interest in taking leadership for systems change.<br><br>Evidence of little or no consistent communication with school staff and families.<br><br>Is uncomfortable, unskilled, or unwilling to be a compelling spokesperson for BAM Program.
